# Coronary Flow Velocity Reserve by Stress Echocardiography: The Prognostic Key of Multiple Heart Diseases

**DOI:** 10.3390/diagnostics16193256

**Published:** 2026-10-08

**Authors:** Jorge Lowenstein

**Affiliations:** Servicio Cardiodiagnóstico, Investigaciones Medicas, Buenos Aires C1056ABA, Argentina; lowensteinjorge@hotmail.com

**Keywords:** coronary flow velocity, coronary flow velocity reserve, coronary microvascular dysfunction, echocardiography, prognostic heart diseases

## Abstract

Although evaluating coronary flow velocity reserve (CFVR) via stress echocardiography (SE) has been available for over three decades, its integration into routine clinical practice remains underutilized globally despite substantial technical and conceptual advances. A diminished CFVR serves as a potent predictor of major adverse cardiovascular events (MACE) and all-cause mortality across a broad spectrum of cardiovascular diseases. Its prognostic value is particularly robust in epicardial coronary artery disease, heart failure, coronary microvascular dysfunction, nonischemic cardiomyopathies, metabolic disorders (e.g., diabetes mellitus and obesity), hypertrophic cardiomyopathy, systemic autoimmune diseases, and cardiac allograft vasculopathy. This comprehensive narrative review details the technical methodology for interrogating resting and stress-induced coronary flow velocity (CFV) under physical and pharmacological protocols. Furthermore, it highlights the clinical applicability of CFVR by synthesizing single-center observational insights with evidence from large-scale multicenter registries, advocating for its broader adoption into diagnostic workflows to refine risk stratification and guide therapeutic management.

## 1. Introduction

Evaluating coronary flow velocity reserve (CFVR)—a key quantitative index reflecting maximal microvascular vasodilator capacity—yields essential insight into the functional integrity of the coronary microcirculation [1]. Consequently, CFVR reflects not only the hemodynamic severity of epicardial stenosis but also the broader structural and functional alterations imposed by various cardiomyopathies on the microvascular bed. Noninvasive interrogation of coronary hemodynamics via transthoracic Doppler echocardiography (TTDE), specifically targeting the distal left anterior descending artery (LAD), was pioneered in the late 1990s by European and Japanese investigator groups [2,3]. Over the past decades, this modality has gained clinical traction due to its noninvasive nature, high feasibility, and favorable safety profile. Recent advances in ultrasound technology—including high-frequency matrix-array transducers, enhanced digital signal processing, and high-sensitivity color Doppler—have expanded diagnostic capabilities beyond the mid-to-distal LAD [4,5]. Today, reliable flow velocity recordings can also be obtained within the right coronary artery (RCA) and left circumflex (LCx) territories [6]. Hyperemic responses, elicited via physical or pharmacological stress, manifest as elevated spectral Doppler velocity profiles; CFVR is subsequently calculated as the ratio of hyperemic peak diastolic velocity to baseline peak diastolic velocity, with values ≥ 2.0 historically defining intact reserve. In epicardial coronary disease, CFVR offers an objective metric to confirm or exclude hemodynamically significant lesions proximal to the interrogation site. Unlike qualitative regional wall motion analysis, CFVR yields a reproducible, quantifiable parameter that strengthens diagnostic accuracy. Beyond obstructive atherosclerosis, the prognostic impact of impaired CFVR has been validated as an independent determinant of long-term survival across diverse cardiovascular cohorts in major multicenter registries [7]. This in-depth narrative review outlines the technical execution of TTDE-derived CFVR and evaluates its growing clinical utility as a key risk-stratification tool across a broad spectrum of cardiac conditions. Literature selection was non-systematic, reflecting extensive personal experience in transthoracic coronary flow assessment combined with landmark studies, foundational evidence, and relevant guidelines across the various clinical scenarios addressed.

### 1.1. First Step: Baseline Coronary Flow Analysis

State-of-the-art practice requires assessing baseline CFV as a preliminary step before measuring CFVR. Determining velocities rather than volumetric flows is preferred, as measuring coronary artery diameter is technically challenging; nevertheless, both yield equivalent clinical information. Angle correction is unnecessary because CFVR represents a ratio between hyperemic and baseline flow velocities; however, the interrogation angle must be kept as small as possible (ideally below 20°).

A high-frequency transducer (4–7 MHz) could be used to visualize the mid-to-distal left anterior descending (LAD) artery, given that the distance between the chest wall and the distal LAD is under 6 cm in adults. Conversely, the right coronary artery (RCA) and left circumflex (LCx) artery lie deeper and require lower-frequency transducers for optimal visualization. Today, a single 4-to-5 MHz matrix (multifocus) transducer is commonly employed for all three main coronary arteries. For color Doppler flow mapping, the velocity scale should initially be set at approximately 20 cm/s and subsequently adjusted for optimal image clarity.

### 1.2. Approaches to Localizing the Coronary Arteries

Several diagnostic approaches exist for localizing the mid-to-distal LAD:

- Approach A: With the patient in the left lateral decubitus position, low parasternal long-axis views from the fourth or fifth intercostal space are used to exclude the right ventricle and depict the anterior interventricular sulcus.
- Approach B: Starting from a modified apical three-chamber view, the operator can track more distally along the vessel. The key technique involves identifying the interventricular groove, which requires successfully excluding the right ventricle.
- Approach C: On occasion, CFV is most easily measured from a parasternal short-axis view, where the mid-to-distal LAD is identified higher up in the anterior interventricular sulcus. Once arterial flow is visualized as a rounded diastolic image, the transducer is rotated to obtain the optimal long-axis view of the artery.

The posterior descending artery (PDA), a branch of the RCA, is imaged from a modified apical two-chamber view using counterclockwise rotation and anterior probe angulation. The mid-to-proximal segment of the obtuse marginal (OM) branch of the LCx is imaged from a modified apical four-chamber view with 50–80° clockwise rotation and posterior probe angulation.

### 1.3. Doppler Velocity Measurements and Technical Feasibility

CFV is measured using pulsed-wave Doppler at frequencies ranging between 1.75 and 3.5 MHz. The mid-to-distal segment of the LAD artery is visualized as a tubular color signal (2–4 mm in length) within the anterior interventricular groove, with flow directed toward the apex and transducer (Figure 1). Flow velocity is quantified by positioning a 2–3 mm sample volume directly over the color Doppler signal, yielding a characteristic biphasic spectral waveform defined by a smaller systolic velocity component (mean: 8–16 cm/s) and a predominant, trapezoidal diastolic component (mean: 16–32 cm/s). (Figure 2). Resting CFV in the LAD naturally decreases as it travels downstream; in healthy individuals, velocities peak in proximal segments and reach their nadir distally [8]. Cardiac motion occasionally hampers complete Doppler spectral envelope recording throughout the entire cardiac cycle; as a result, TTDE routinely measures only the diastolic component. With modern technology and modest training—comparable to the ~100 cases required to interpret resting wall motion abnormalities under supervision—TTDE evaluation of LAD CFVR can be successfully performed in nearly all patients. This approach rarely requires ultrasound-enhancing agents, even in the obese population [9]. Failing to locate at least one segment of the mid-to-distal LAD is exceptional; however, even trained operators can locate the PDA in only about 75% of patients. Detecting LCx flow remains considerably more technically demanding, with feasibility rates limited to approximately one-third of cases (Figure 3, Table 1).

### 1.4. Clinical Significance and Stenosis Detection

Assessing coronary flow characteristics during routine echocardiography provides critical insights into abnormal coronary physiology. Elevated resting CFV, independent of reduced CFVR, predicts worse survival in patients with chronic coronary syndromes and preserved resting global left ventricular function. During a median follow-up of 5.9 ± 4.3 years, 1121 patients (15%) died. Multivariable analysis identified a resting CFV threshold of ≥32 cm/s via receiver operating characteristic curve analysis as optimal, showing an independent association with mortality (hazard ratio [HR], 1.24 [95% CI, 1.10–1.40]; *p* < 0.0001) [22]. Various metabolic, myocardial, hydraulic, microvascular, and sympathetic factors converge to produce this phenotype of elevated resting CFV. Color Doppler interrogation along the vessel length enables detection of localized aliasing or turbulence. At stenosis sites, coronary flow accelerates to compensate for luminal narrowing; velocities exceeding 1 m/s strongly point to significant stenosis. Furthermore, comparing proximal left vessel velocity to distal LAD flow yields high sensitivity and specificity for detecting significant obstruction when proximal CFV doubles distal values [23]. Across any segment of the coronary tree, a 100% velocity increase between adjacent segments suggests significant focal stenosis (Figure 4). The systolic component of CFV may be preserved or increased distal to an occlusion in severe LAD stenosis. Daimon et al. reported that a mean diastolic-to-systolic velocity ratio cutoff of 1.5 yielded 81.8% sensitivity and 85.7% specificity for predicting reversible perfusion defects, using thallium-201 single-photon emission computed tomography (SPECT) as the reference standard [24]. Similarly, our group demonstrated 85.7% sensitivity and 84.2% specificity using a peak diastolic-to-systolic ratio cutoff of 1.6 against invasive coronary angiography.

### 1.5. Acute Myocardial Infarction Assessment

Evaluating resting CFV patterns following acute myocardial infarction (AMI) is highly feasible via TTDE. Systolic flow reversal is a straightforward, reliable marker for predicting non-recovery of left ventricular function in anterior infarctions treated with primary reperfusion. In patients achieving TIMI 3 flow, LAD signals display larger color flow areas and higher velocities than in those with TIMI 1 or 2 flow. A rapid deceleration time of the diastolic CFV envelope (<600 ms) serves as another robust indicator of poor functional recovery and adverse prognosis following primary angioplasty in AMI. Thus, simple non-invasive parameter measurements in the reperfused infarct-related artery offer reliable early risk stratification. Finally, total LAD occlusion is characterized by a complete absence of color Doppler flow. Retrograde diastolic flow distal to a total occlusion reflects collateral flow supplying the ischemic myocardium, which is readily demonstrable on TTDE [25] (Figure 5).

### 1.6. Diagnostic Artifacts and Pitfalls

Coronary blood flow can occasionally be mistaken for pulmonary insufficiency—A rare error easily avoided with basic operator training. Internal mammary or intercostal arterial flows are readily distinguished by their systolic velocity predominance, characteristic of systemic arteries (Figure 6). Other neighboring structures present distinct Doppler features:

- Coronary Venous Flow: Features prominent systolic flow reversal (Figure 6).
- Intercostal Veins: Display marked respiratory variation, with flow attenuating or disappearing during the Valsalva maneuver (Figure 6).
- Anterior Pericardial Space**:** May exhibit heterogeneous flow, but lacks the characteristic biphasic coronary velocity profile (Figure 6).

While misidentifying the LAD as a parallel diagonal branch or nearby vessel is theoretically possible, it is uncommon in practice. Similarly, the PDA can occasionally be confused with right ventricular inflow; however, the trapezoidal coronary flow envelope is easily distinguished from normal double-peaked diastolic inflow waves.

### 1.7. Second Step: Analysis of CFVR During Stress

CFVR can be evaluated using various stressors. While treadmill exercise is unsuitable for Doppler acquisition and semi-supine ES presents technical challenges, vasodilator stress is the most practical choice. It simplifies the protocol and optimizes the Doppler signal, making the coronary flow signal larger, clearer, and brighter during hyperemia [9]. Vasodilators such as adenosine and dipyridamole act directly on the coronary microcirculation. In contrast, dobutamine and exercise induce secondary vasodilation mediated by increased myocardial oxygen demand and endogenous adenosine release. Direct vasodilators remain the clinical standard because they achieve greater microvascular recruitment, maximally increasing coronary flow velocity. Nevertheless, Meimoun et al. In an elegant study demonstrated a strong correlation and concordance between dobutamine- and adenosine-derived CFVR across a wide spectrum of LAD disease. Their findings establish dobutamine as a reliable alternative to adenosine—particularly in patients with contraindications to adenosine or those already undergoing dobutamine stress echocardiography (DOBSE) [26]. Compared to adenosine, dipyridamole is better tolerated, causes less hyperventilation, yields superior image quality, and is more cost-effective in certain healthcare systems. Furthermore, its prolonged duration of action allows for nearly simultaneous assessment of wall motion and coronary flow within a single acquisition. Our group has also validated a rapid adenosine protocol utilizing a single 6 mg intravenous bolus, completing the assessment in under one minute. In patients with equivocal wall motion findings after DOBSE or ESE, this protocol demonstrated 88.8% sensitivity and 84.6% specificity for detecting significant CAD in the LAD territory. Although exercise CFVR was historically considered unfeasible, recent technical refinements have increased success rates to approximately 80% [27]. Finally, despite extensive data supporting the safety of CFVR measurement across diverse clinical scenarios, testing should strictly be performed in clinically stable patients. In our laboratory, all patients provide written informed consent prior to stress echocardiography with CFVR assessment.

## 2. CFVR During Dipyridamole Stress Echocardiography

Evaluating regional wall motion at baseline and during pharmacological stress [9,28,29,30] remains the predominant diagnostic methodology for coronary artery disease (CAD) in echocardiography laboratories. Dipyridamole stress echocardiography (DIPSE) is a highly effective diagnostic modality, primarily characterized by its low technical complexity, favorable safety profile, and outstanding specificity. However, its main constraint lies in its lower sensitivity among patients with single-vessel disease, particularly when tested under anti-ischemic medical therapy and without concomitant atropine administration.

### 2.1. Patient Preparation and Protocol

Patients fasted for a minimum of 4 h prior to the procedure. Individuals on chronic treatment with dipyridamole-containing compounds (or the novel oral antiplatelet agent ticagrelor) must withhold these medications for at least 24 h before a vasodilator stress test to avoid unpredictably elevated baseline adenosine levels. Similarly, theophylline and caffeine derivatives require a 24 h wash-out period to keep adenosine receptors unblocked. Resting diastolic CFV was measured in the LAD. The Doppler sample volume was positioned as far distally as attainable, ensuring it remained outside high-velocity areas during vasodilator infusion to prevent misinterpreting hyperemic flow dynamics as intrinsic vessel obstructions. Following baseline acquisition—and keeping both the transducer position and Doppler sample volume fixed—dipyridamole was administered at 0.21 mg/kg/min over 4 to 6 min. Flow velocities were continuously monitored and recorded throughout the infusion and for one minute post-completion (Figure 7 and Figure 8). The CFVR index was calculated by dividing peak hyperemic diastolic velocity by the baseline value (Figure 9), with an abnormal result defined as a ratio under 2. Blood pressure and heart rate were automatically measured every 2 min until test completion. Experienced echocardiographers offline-analyzed all parameters, calculating the mean across 3 consecutive cardiac cycles.

### 2.2. Image Acquisition and Optimization

Two-dimensional images were digitized at rest, at 8 and 12 min following the start of dipyridamole infusion, and at minute 14 (after antidote administration). In negative studies, patients without contraindications such as glaucoma or prostatic disorders received 1 mg of atropine at minute 10. Additionally, all non-ischemic DIPSE studies were sensitized using continuous isometric exercise during the minute preceding atropine delivery. Aminophylline (240 mg) was routinely administered at test termination (minute 13), or earlier if clear ischemic wall motion abnormalities emerged.

### 2.3. Diagnostic and Prognostic Value

To enhance DIPSE sensitivity and uncover false-negative results, we integrated CFVR measurements alongside conventional wall motion analysis, as dipyridamole allows simultaneous evaluation of both parameters. In our cohort, combining LAD CFVR measurement with wall motion analysis boosted diagnostic sensitivity from 68.8% to 86.8% [32]. Representative cases showing normal and abnormal LAD CFVR alongside normal wall motion are illustrated in Figure 10. TTDE achieved a 95% feasibility rate for obtaining mid-distal LAD CFVR during DIPSE (Table 1). Rather than providing redundant data, CFVR offers incremental and complementary value to 2D regional wall motion assessment. Furthermore, incorporating CFVR offsets key limitations of functional stress markers, including subjective reading variability and suboptimal sensitivity. Its ease of measurement and interpretation makes CFVR an optimal adjunct to standard 2D echocardiographic findings.

### 2.4. Risk Stratification

Multiple studies confirm the independent prognostic power of pulsed Doppler-derived CFVR during DIPSE. Rigo et al. and Arbucci et al. (the latter from our institution) showed that impaired CFVR provides incremental risk stratification following stress testing [33,34]. While an inducible wall motion abnormality inherently signals an adverse prognosis, assessing CFVR isolates a sub-cohort at heightened risk. Conversely, normal wall motion defines a low-risk subset; nevertheless, among patients without dyssynergy while on medical therapy, abnormal CFVR values correlate with less favorable long-term outcomes [34]. A 2020 multicenter trial in 2149 patients established that blunted heart rate reserve (HRR) during DIPSE reflects cardiac autonomic dysfunction and portends poorer survival. While a patient without wall motion abnormalities sits at intermediate risk, combining this result with preserved CFVR shifts them to a low-risk category, and adding a normal HRR places them in a very low-risk category [35]. Thus, a completely negative test (normal wall motion, CFVR, and HRR) represents the optimal scenario with excellent long-term outcomes, whereas close clinical surveillance is indicated for patients with normal wall motion but impaired CFVR [34]. In a recent, large prospective international outcome trial validating the ABCDE-SE protocol, researchers confirmed that wall motion negativity alone is insufficient to classify patients with known or suspected chronic coronary syndrome as low risk. Incorporating contractile reserve, B-lines, HRR, and particularly CFVR is critical for accurate survival prediction [36]. Notably, ongoing anti-ischemic medication at the time of evaluation does not diminish the prognostic accuracy of CFVR.

### 2.5. Limitations and Future Directions

A major limitation across existing TTDE literature is the exclusive focus on the LAD, as technical success rates for interrogating the RCA and LCx remain suboptimal (Figure 3, Table 1). However, emerging TTDE research evaluating multi-vessel CFVR across all three epicardial territories has yielded promising results [37]. Finally, clinicians should note that a blunted CFVR cannot definitively differentiate severe epicardial stenosis (macrovascular CAD) from microvascular dysfunction. Nevertheless, integrating the overall clinical context, concurrent wall motion abnormalities, or markedly depressed CFVR values can assist in distinguishing between these two entities (Table 2) maintained throughout the infusion, while blood pressure is recorded at baseline and at 1 min intervals following initiation. Adenosine is generally well-tolerated, producing minimal to no significant changes in baseline hemodynamic variables.

### 2.6. CFVR with Adenosine

Adenosine is the primary pharmacological agent used to measure CFVR because it induces maximal arteriolar vasodilation with minimal to no effect on the epicardial arteries. The use of adenosine for CFVR assessment is standard practice in Europe and Japan. The most widely utilized protocol involves a continuous intravenous infusion of 0.14 mg/kg/min over two minutes. Spectral Doppler signals in the distal LAD are recorded at baseline and during peak hyperemia, ensuring the transducer position remains entirely unchanged throughout drug administration. CFV measurements are subsequently analyzed off-line by experienced echocardiographers. Continuous electrocardiographic and heart rate monitoring is required.

### 2.7. CFVR During Dobutamine Stress Echocardiography

DOBSE is currently one of the most widely used pharmacological stress modalities in stress echocardiography laboratories worldwide. Although evaluating CFVR during DOBSE is technically more demanding than with dipyridamole or adenosine, its feasibility in the LAD territory is similarly high (Table 1). Briefly, dobutamine is administered via an infusion pump to assess CFVR without microbubble contrast agents. The infusion is initiated at 5 µg/kg/min and titrated in 3 min stages to 10, 20, 30, 40, and 50 µg/kg/min, or until the target heart rate is achieved. Atropine (up to 1 mg) may be co-administered during the final 50 µg/kg/min stage—or in fractional doses of 0.25 mg across the final four stages—to achieve the target heart rate (Figure 11). Patient symptoms and hemodynamic variables, including heart rate and blood pressure, are recorded at each stage. As our group has previously demonstrated, recording CFVR at every stage of the test is unnecessary. It can be reliably determined at baseline and during high-dose dobutamine infusion, provided the heart rate increases by at least 50 bpm above baseline or reaches 75% of the predicted maximum value [15]. CFVR is calculated as the ratio of peak diastolic flow velocity to baseline diastolic flow velocity, with final values representing an average across three consecutive cardiac cycles. Consistent with prior invasive and non-invasive literature, an abnormal CFVR is defined as a value < 2.0 (Figure 9). In our experience, CFVR during DOBSE can only be reliably measured within the LAD territory due to excessive translation and cardiac motion during beta-adrenergic-induced tachycardia. Representative examples of normal and abnormal studies are illustrated in Figure 12.

### 2.8. Clinical Evidence and Prognostic Value

In 2003, M. Takeuchi et al. (Osaka) demonstrated the feasibility of measuring LAD-CFVR during DOBSE, showing diagnostic accuracy equivalent to wall-motion analysis for detecting LAD stenosis. Notably, CFVR provided incremental sensitivity in patients with poor acoustic windows or inconclusive wall-motion results [16]. In 2011, Lowenstein, Tiano et al. investigated the incremental prognostic value of LAD CFVR among patients with normal contractile responses during pharmacological stress echocardiography (dobutamine up to 50 µg/kg/min [n = 164] or dipyridamole at 0.84 mg/kg over 4 min [n = 185]) [38]. In multivariable logistic regression analysis, CFVR emerged as the sole independent predictor of combined hard events (OR: 4.03; 95% CI: 1.82–8.92; *p* = 0.0006) (Figure 13). Overall, CFVR proved to be a robust, independent prognostic indicator:

- Normal CFVR (≥2.0): Associated with an annual hard event rate (acute myocardial infarction, coronary artery bypass grafting, or cardiovascular death) of 1% per year.
- Abnormal CFVR (<2.0): Associated with a 4% annual incidence of hard events over a mean follow-up period of 46 ± 24 months.

Furthermore, a graded risk stratification of hard events was observed based on the severity of CFVR reduction:

- CFVR ≥ 2.0: Reference event rate (OR: 1.08);
- CFVR 1.65 to 1.99: OR = 3.40;
- CFVR 1.47 to 1.64: OR = 4.57;
- CFVR < 1.47: OR = 5.06 (*p* = 0.02) (Table 3).

Importantly, simultaneous visual assessment of regional wall motion during stress echocardiography was entirely normal across all these patients. The authors concluded that a reduced CFVR in patients meeting normal contractility criteria identifies a subgroup with a significantly worse mid- to long-term prognosis, irrespective of whether dobutamine or dipyridamole was administered [38]. These findings have since been validated by larger multicenter trials, underscoring the clinical rationale for systematically integrating LAD-CFVR into all stress echocardiography protocols [7,27,39,40,41].

### 2.9. CFVR During Exercise Stress Echocardiography

ESE is an essential clinical tool that delivers valuable diagnostic and prognostic insight. It remains the most widely utilized stress modality because it is physiological, straightforward, and safe. The recommended initial workload is 25 W, increased by 25 W every 2 min until standard clinical or electrocardiographic endpoints are reached. Continuous 12-lead ECG and periodic blood pressure monitoring are maintained throughout the study. Echocardiographic images are acquired at rest and systematically throughout exercise. Post-stress images, matching baseline views, are acquired immediately following peak exertion. Anatomical mapping of the coronary vessels is performed using color Doppler. The color Doppler velocity scale is initially set to ~0.20 m/s and dynamically adjusted to optimize vessel visualization. A pulsed-wave Doppler sample volume is then aligned as parallel as possible to blood flow. CFV signals are recorded at rest and during exercise within the exact same arterial segment. Following baseline measurements, flow velocity is reassessed once the patient reaches a heart rate of ~100 bpm, which typically yields superior signal quality compared to higher heart rates. If flow velocity has not doubled at this stage, monitoring continues until the protocol is complete (Figure 14). CFVR is calculated as the ratio of peak exercise flow velocity to baseline resting flow velocity. In routine clinical practice, a CFVR cutoff value ≥ 1.9 is considered normal [27]. Unlike pharmacological stress, exercise-induced hemodynamic changes—such as increased baseline heart rate and shortened diastolic duration—typically result in slightly lower peak-to-baseline flow ratios, justifying our empirically derived threshold of <1.89 over the traditional pharmacological cutoff of <2.0. Furthermore, Zagatina et al. demonstrated that measuring CFVR during supine bicycle exercise is highly feasible, as normal hyperemic responses are typically achieved prior to reaching submaximal target heart rate.

### 2.10. CFVR in Different Clinical Scenarios

Based on our single-center experience in Buenos Aires since 1998, combining LAD CFVR with left ventricular wall motion analysis represents the optimal echocardiographic approach for evaluating the flow-function relationship. Table 4 outlines the various clinical scenarios where baseline flow dynamics and CFVR assessments provide critical diagnostic and prognostic value.

### 2.11. Diagnosis and Prognosis of Significant Coronary Stenosis

CFVR is widely utilized to diagnose CAD. The LAD is easily accessible, allowing noninvasive CFVR measurement without contrast agents in almost all patients. An abnormal CFVR is defined as <2.0, consistent with invasive and noninvasive studies establishing this threshold as predictive of myocardial ischemia. TTDE-derived CFVR in the LAD—and other coronary vessels—correlates closely with invasive Doppler wire measurements and compares favorably with nuclear imaging, SE, and myocardial contrast echocardiography. Inter- and intraobserver variability for TTDE-CFVR are low, remaining below 5% in published literature. Consequently, CFVR facilitates CAD identification and reliably rules out the functional significance of epicardial stenosis. In 2001, we presented our early experience at a European Society of Cardiology congress in Stockholm (“Noninvasive assessment of CFVR by TTDE in a general referral population: experience in 957 patients”). Peak diastolic CVF was continuously monitored via pulsed-wave Doppler in the distal LAD at rest and during hyperemic stress induced by dipyridamole (0.84/mg/kg over 4 min; 775), adenosine (014/ mg/kg/min over 2 min: 104), or dobutamine (up to 50 ug kg/min 78 patients). CFVR was correlated with coronary angiography performed within one week. Demonstrating 88.8% sensitivity and 74.0% specificity for detecting critical LAD stenosis, these data confirmed the utility of TTDE in routine clinical practice [42]. Over the past decade, numerous studies have validated and expanded upon these initial findings, extending noninvasive CFVR measurement to other coronary territories [7,8,9,22,36]. Large landmark studies have established that reduced CFVR independently predicts adverse cardiac events in obstructive CAD, adding incremental prognostic value to anatomic stenosis severity and traditional wall motion analysis. Recently, Cortigiani et al. evaluated 749 patients with chronic coronary syndromes and ≥ 50% LAD stenosis, demonstrating that CFVR-guided revascularization improved 10-year survival regardless of inducible wall motion abnormalities (WMA) [43]. Notably, patients with isolated CFVR reduction (<2) without inducible WMA who underwent revascularization derived a 10-year survival benefit comparable to those presenting with both impaired CFVR and inducible ischemia (76% vs. 53% relative to conservative management (*p* < 0.0001). This directly challenges the traditional paradigm of guiding revascularization solely by inducible ischemia [43]. Occasionally, a discordance between normal CFVR and angiographically significant stenosis occurs. Potential explanations include:

1. Well-developed collateral circulation mitigating severe stenosis downstream;
2. Angiographic overestimation by visual inspection;
3. Proximal CFVR measurement relative to the stenosis (run-off phenomenon);
4. Misdirected sampling of adjacent parallel vessel branches.

Distinguishing epicardial stenosis from microvascular dysfunction remains a key limitation of isolated CFVR. However, in our experience, severe impairment (CFVR < 1.6) or concomitant wall motion dyssynergies strongly point toward obstructive epicardial CAD. Thus, the integrated assessment of coronary flow, flow reserve, and myocardial contractility represents a comprehensive tool for clinical decision-making (Table 2). Ongoing research is currently evaluating whether combining myocardial work index with CFVR enhances risk stratification compared to global longitudinal strain or CFVR alone.

### 2.12. Follow-Up After Coronary Angioplasty, Stenting, and Revascularization

Severe LAD stenosis markedly impairs TTDE-derived CFVR. While pre-intervention values are typically severely blunted, they normalize rapidly following successful stent deployment [44]. Performing TTDE in the days following LAD stenting avoids the procedural risks of intracoronary Doppler, serving as an effective, noninvasive approach to evaluate acute revascularization results.

### 2.13. Monitoring Restenosis Across Coronary Territories

Mid- to long-term CFVR values < 2.0 (induced by adenosine or dipyridamole) exhibit high sensitivity (80–100%) and specificity (80–100%) for detecting restenosis > 70% in the LAD. Beyond the LAD, noninvasive feasibility for vasodilator CFVR assessment has been documented as 95% in the LAD, 85% in the RCA, and 81% in the LCx [19]. These findings demonstrate that TTDE-CFVR is a versatile, accurate tool for serial surveillance of restenosis across all major coronary arteries following percutaneous coronary intervention.

### 2.14. Evaluation of Left Internal Mammary Artery (LIMA) Grafts

The feasibility of TTDE for evaluating patients with LIMA grafts is remarkably high (80–100%). Occasionally, the precise anastomotic site with the native artery can be visualized, enabling direct assessment of graft dysfunction.

Flow Dynamics and Evaluation Indices

- Normal Function: Systolic flow in the native LIMA decreases near the anastomosis, whereas diastolic flow increases significantly. The normal systolic-to-diastolic peak velocity ratio is approximately 0.5 ± 0.2.
- Graft Dysfunction: In the presence of graft dysfunction, this ratio increases significantly to 3.4 ± 0.7.
- Complete Occlusion: When the graft is completely occluded, LIMA blood flow resembles that of a standard systemic artery.

Measuring CFVR in the distal LAD is more clinically relevant than baseline examination alone, as a normal result effectively rules out significant restenosis. However, LIMA CFVR does not reflect downstream LAD perfusion in the same manner as direct LAD CFVR measurement. Overall, assessing LAD CFVR using TTDE is a highly effective, noninvasive approach to confirm graft patency after Coronary Artery Bypass Grafting.

### 2.15. Evaluation of Intermediate Coronary Stenosis

Dating back to 2001, our group observed a strong correlation between noninvasive CFVR assessment and LAD stenosis severity. However, in patients with intermediate stenosis (50–70% luminal diameter reduction), CFVR findings were equivocal: 50% demonstrated a preserved CFVR (>2.0), while the remaining 50% had an impaired CFVR (<2.0) [45]. (Figure 15). To evaluate outcomes in deferred versus performed percutaneous coronary intervention based on CFVR in patients with intermediate Fractional Flow Reserve (FFR), data from the ILIAS Registry demonstrated that CFVR serves as a valuable complementary risk-stratification tool for optimizing treatment strategies [46].

### 2.16. Patients with LBBB of Uncertain Etiology

Patients with left bundle branch block (LBBB) exhibit impaired early diastolic flow in the LAD due to increased compressive resistance from delayed left ventricular relaxation. Furthermore, exercise perfusion defects on scintigraphy in these patients correlate with reduced CFVR, indicating microvascular dysfunction within the same vascular territory. However, in the absence of obstructive CAD, CFVR abnormalities linked to LBBB are typically subtle or mild. Conventional noninvasive diagnostic modalities have well-known limitations in detecting CAD among patients with LBBB. Nevertheless, advancements in TTDE now allow for precise noninvasive CFVR measurement. In a landmark study, Cortigiani et al. demonstrated that an abnormal LAD-CFVR is a strong, independent predictor of mortality and composite endpoints (death or myocardial infarction) in LBBB patients. Crucially, this prognostic value persists even in patients with a negative stress echocardiogram on medical therapy [47]. Additionally, a 2024 systematic review highlighted the clinical utility of echocardiographic CFVR for risk stratification in LBBB while emphasizing the need for standardized measurement protocols [48].

### 2.17. Evaluation of Microvascular Circulation

In a prospective collaborative study led by Dr. Rodríguez Zanella with active participation from our laboratory, we demonstrated that among 135 patients with ischemia and non-obstructive coronary arteries (INOCA), 26% exhibited a reduced CFVR [49]. In patients with chest pain and angiographically normal coronary arteries, pharmacologic stress echocardiography can identify a subgroup with a less favorable prognosis. Sicari et al. showed that Kaplan–Meier 48-month survival estimates for hard endpoints were significantly higher in patients with normal CFVR and preserved baseline/stress LV function compared to those with abnormal CFVR (96% vs. 55%, *p* = 0.001). Thus, CFVR provides incremental prognostic value beyond clinical and angiographic profiling [50]. Similarly, Al-Amri et al. demonstrated that CFVR was impaired in patients with diabetes, hypertension, and obesity, with greater impairment corresponding to an increasing burden of risk factors. The authors recommended aggressive risk factor modification in these patients even when dobutamine stress echocardiography (DOBSE) is negative for regional wall motion abnormalities [51]. Results from the prospective iPOWER study remained statistically significant in multivariate analysis, showing a hazard ratio (HR) of 1.05 (95% CI: 1.01–1.09, *p* = 0.01) for adverse cardiovascular events per 0.1-unit decrease in CFVR. Exploratory analyses also linked low CFVR to higher rates of rehospitalization for angina and all-cause mortality [52]. Furthermore, comparative sex-based analyses revealed that abnormally low CFVR confers a similarly poor prognosis in both men and women, underscoring that microvascular impairment predicts adverse outcomes regardless of sex [53]. Finally, a systematic review and meta-analysis by Kelshiker et al. concluded that reduced CFVR is strongly linked to an increased risk of all-cause mortality and major adverse cardiovascular events (MACE) across diverse etiologies. An abnormal CFVR predicted adverse outcomes in multiple cohorts, including patients with isolated coronary microvascular dysfunction (CMD), where it carried a mortality HR of 5.44 (95% CI: 3.78–7.83) and a MACE HR of 3.56 (95% CI: 2.14–5.90). These findings support recent guidelines recommending routine coronary flow assessment to identify high-risk individuals who benefit from targeted vascular risk management [39].

### 2.18. CFVR in Valvular Diseases

CFVR assessment in valvular disease identifies CMD that develops independently of epicardial coronary artery stenosis. Combining regional wall motion abnormalities with CFVR provides enhanced risk stratification. This approach is particularly valuable in patients with discordant findings, where impaired CFVR in the absence of wall motion abnormalities indicates isolated microvascular disease [7]. CFVR is frequently blunted in valvular heart disease, with the degree of impairment directly reflecting disease severity. Pioneering TTDEE work by Akasaka et al. established that chronic mitral regurgitation significantly depresses CFVR compared with controls (2.1 ± 0.5 vs. 3.3 ± 0.6, *p* < 0.01). This reduction is driven by an elevated resting CFV (28 ± 8 vs. 19 ± 6 cm/s, *p* < 0.01), whereas maximal hyperemic flow velocity remains largely preserved. The primary determinant of restricted CFVR is increased left ventricular (LV) end-diastolic pressure (r2 = 0.49, *p* < 0.0001), along with secondary correlations to LV mass index, LV end-diastolic volume, and regurgitant volume; notably, this impairment improves following mitral valve surgery [54]. Similarly, CFVR is markedly reduced in severe aortic stenosis despite angiographically normal coronary arteries. This dysfunction stems from a mismatch between reduced myocardial supply (due to lower coronary perfusion pressure) and heightened metabolic demand (from increased LV workload). However, evidence suggests that CFVR impairment correlates more strongly with aortic valve area and peak gradient than with the extent of LV hypertrophy. The long-term prognostic value of CFVR for predicting cardiovascular morbidity and mortality was demonstrated over a 9-year follow-up in patients with aortic stenosis, where CFVR emerged as an independent predictor of adverse events [55]. Corroborating these findings, successful aortic valve intervention significantly increases coronary flow. TAVI, for instance, yields a 35% increase in CFVR and a 39% increase in microvascular resistance reserve at 6 months—primarily driven by a reduction in baseline coronary flow—though this represents only partial restoration of microvascular function [56].

### 2.19. Differential Diagnosis and Prognosis of Dilated Cardiomyopathy

Reduced CFVR is a powerful independent predictor of adverse outcomes in dilated cardiomyopathy (DCM), including overall mortality, major cardiac events, and worsening heart failure. By quantifying CMD, CFVR offers incremental prognostic value over traditional clinical and echocardiographic parameters. In a landmark study of 129 DCM patients by Rigo et al., an abnormal CFVR independently predicted survival (HR 4.0, 95% CI: 1.1–15.6). At 75 months, event-free survival was strikingly lower in patients with impaired CFVR compared to those with preserved reserve (22% vs. 70%, *p* < 0.0001) [57]. Furthermore, preserved CFVR in DCM correlates with favorable LV remodeling and better functional response to cardiac resynchronization therapy (CRT)—serving as the sole independent predictor of 6-month LV functional recovery in 40 CRT recipients [58]—whereas blunted CFVR portends worse outcomes and higher ventricular arrhythmia inducibility.

### 2.20. Coronary Flow Reserve in Hypertrophic Cardiomyopathy

In hypertrophic cardiomyopathy (HCM), angina and myocardial ischemia frequently occur without epicardial coronary stenosis. Microvascular dysfunction is a recognized hallmark and strong independent predictor of clinical deterioration and death in HCM, often preceding overt clinical decline by years even in mildly symptomatic or asymptomatic individuals. During DIPSE with normal wall motion, Cortigiani et al. observed that mean CFVR was markedly lower in HCM patients than in controls (2.12 ± 0.39 vs. 2.78 ± 0.58, *p* < 0.0001). Blunted CFVR was present in 67% of symptomatic versus 17% of asymptomatic patients (*p* < 0.0001) and was significantly more pronounced in those with left ventricular outflow tract obstruction. Over 3 years of follow-up, adverse event rates were substantially higher in patients with abnormal CFVR (79% vs. 17%, *p* < 0.0001), reaffirming CFVR as an independent prognostic marker in HCM [59]. More recently, data from Belgrade, Serbia, confirmed that HCM patients with preserved LAD-CFVR achieved superior cumulative event-free survival compared to those with impaired LAD-CFVR (96.4% and 90.9% vs. 66.9% and 40.0% at 5 and 8 years, respectively; log-rank 37.2, *p* < 0.001). Impaired LAD-CFVR (<2.0) stands as a robust predictor of adverse cardiac events. Crucially, when evaluating LAD-CFVR for risk stratification, additional assessment of RCA provides no additive diagnostic or prognostic value [60].

### 2.21. Coronary Flow Reserve in Patients with Heart Failure

In congestive heart failure (HF), CFVR is characteristically restricted. This attenuation is driven by elevated resting baseline flow velocities, tachycardic heart rates, and increased LV preload and volumes [61]. In a multicenter cohort of 610 non-ischemic HF patients, abnormal CFVR (<2.0) was detected in 29% of cases and independently predicted adverse clinical outcomes [62]. Identifying blunted CFVR pinpoints a high-risk cohort and highlights CMD as both a central disease marker and a target for therapy across the entire spectrum of ejection fractions. Ultimately, incorporating CFVR overcomes the diagnostic constraints of relying solely on regional wall motion assessment, offering critical insights in HF where microvascular pathology develops independently of epicardial coronary artery disease [7].

### 2.22. Coronary Flow Reserve and Heart Transplantation

Cardiac allograft vasculopathy (CAV) is the primary factor limiting long-term success in heart transplantation. While traditional techniques such as coronary angiography, intravascular ultrasound (IVUS), multislice CT, and intracoronary Doppler guidewires provide undeniable value, they are not without potential complications. Furthermore, these invasive methods are less likely to be available in daily clinical practice, especially for the longitudinal follow-up of patients. SE using pharmacological agents or exercise is the most commonly utilized non-invasive technique for monitoring heart transplant recipients. CFVR measured by TTDE holds strong prognostic value in heart transplant recipients, predicting MACE and all-cause mortality. Thus, CFVR serves as a powerful non-invasive marker for CAV and long-term outcomes. In a study of 98 heart transplant patients followed for a median of 3.3 years, a low CFVR (<2.0) was associated with significantly reduced MACE-free survival (HR 4.3, 95% CI 2.2–8.4, *p* < 0.0001) and increased all-cause mortality (HR 4.7, 95% CI 2.0–11.3, *p* < 0.0001) compared to patients with preserved CFVR (≥2.0). When combined with left ventricular global longitudinal strain (GLS), CFVR provided incremental prognostic value even after adjusting for CAV severity and time since transplantation [63]. An earlier landmark study of 66 heart transplant patients contributed significantly to this field, demonstrating that a shorter deceleration time of diastolic flow velocity (≤840 ms) and a lower CFVR (≤2.6) are reliable markers for CAV. In that study, assessments were performed using contrast-enhanced TTDE during adenosine infusion at a rate of 0.14 mg/kg. CFVR emerged as the primary independent predictor of MACE during a follow-up period of 19 ± 5 months [64]. CFVR is an ideal tool for this patient population due to its non-invasive nature, low cost, lack of ionizing radiation, and high repeatability. Given its strong independent prognostic value and risk-stratification capability, serial CFVR assessment by TTDE may be considered part of routine non-invasive CAV surveillance in heart transplant recipients.

### 2.23. CFVR in Rheumatic Diseases

CFVR is significantly impaired in patients with rheumatic diseases and psoriatic arthritis, reflecting the CMD that occurs even in the absence of obstructive coronary artery disease. This impairment represents an important marker of subclinical cardiovascular disease and carries prognostic significance for adverse cardiovascular outcomes. A meta-analysis of 21 studies including 709 patients with rheumatic diseases demonstrated significantly lower CFVR values compared to healthy controls (standardized mean difference = −1.51, 95% CI −1.91 to −1.11; *p* < 0.001). This impairment was independent of traditional cardiovascular risk factors, age, lipid levels, body mass index, blood pressure, or inflammatory burden. The severity of CFVR impairment varies by disease type. Patients with predominantly autoimmune features, such as systemic lupus erythematosus, showed significantly lower CFVR compared to those with mixed autoimmune and autoinflammatory features, such as psoriatic arthritis. This suggests that different pathogenic mechanisms influence the degree of myocardial microvascular dysfunction (MCD) [65]. This supports the role of systemic inflammation in MCD and suggests that the coronary microcirculation may represent an extracutaneous site involved in the immune-mediated injury of psoriasis. Consequently, we should actively search for and diagnose CMD in patients with severe psoriasis [66]. The severity of CFVR impairment correlates with disease activity: patients with abnormal CFVR (≤2.5) had higher Psoriasis Area Severity Index (PASI) scores, and the PASI score remained the only independent determinant of abnormal CFVR in multivariable analysis. In the context of systemic autoimmune and rheumatic diseases—including the meta-analysis a cut-off of <2.5 yields greater sensitivity for identifying early, subclinical coronary microvascular dysfunction and endothelial damage in patients with systemic chronic inflammation, particularly when assessed via non-invasive TTDE In another study of 198 patients with systemic inflammatory disorders (including rheumatoid arthritis, systemic lupus erythematosus, and psoriasis), those in the lowest myocardial flow reserve tertile (MFR < 1.65) had significantly higher all-cause mortality (HR 2.4, 95% CI 1.05–5.4; *p* = 0.038) and lower MACE-free survival (HR 3.6, 95% CI 1.7–7.6; *p* = 0.001) over a median follow-up of 7.8 years [67]. In conclusion, among patients with systemic inflammatory disorders, impaired CFVR is independently associated with worse cardiovascular outcomes and all-cause mortality, regardless of the non-invasive method used.

## 3. Prognostic Value in Other Medical Conditions

### 3.1. Kawasaki Disease and Congenital Heart Diseases

There is evidence demonstrating that TTDE allows for the reliable measurement of CFVR in the LAD artery in children with Kawasaki disease. Non-invasive measurement of CFVR in the proximal LAD using TTDE accurately reflects invasive measurements obtained via the Doppler guidewire method in pediatric patients with various other heart diseases, including ventricular septal defect, patent ductus arteriosus, and tricuspid atresia. CMD could play an important role in the pathophysiology of congenital heart diseases [68]. According to the American Heart Association, CFVR assessment helps identify functional coronary abnormalities and impaired vasodilatory capacity in Kawasaki disease patients during long-term follow-up [69]. Experience with non-invasive CFVR in children is still limited, although growing: most studies come from a small number of centers (mainly Japan), with small sample sizes (typically 20–80 patients), and have focused almost exclusively on Kawasaki disease and a subgroup of congenital heart diseases used as reference or control groups. Broad multicenter validation and standardized age-based pediatric reference values are not yet available, which limits the generalizability of cutoff points to routine clinical practice.

### 3.2. Takotsubo Syndrome

Recent evidence highlights CMD as a key factor contributing to myocardial ischemia and transient left ventricular dysfunction in Takotsubo syndrome. Its etiology appears multifactorial, involving autonomic dysregulation, endothelial dysfunction, inflammation, and myocardial edema, all of which contribute to the transient impairment of myocardial perfusion. Invasive studies consistently demonstrate elevated microvascular resistance and reduced CFVR during the acute phase, followed by a gradual recovery that correlates with improvement in left ventricular function (LV). Furthermore, CMD in Takotsubo syndrome has been associated with an adverse prognosis, including higher mortality and persistent LV dysfunction [70].

### 3.3. Chagas Disease

Chagas disease, caused by *Trypanosoma cruzi*, remains one of the leading infectious causes of non-ischemic cardiomyopathy worldwide. Although an estimated 6–7 million individuals are chronically infected, approximately 20–30% will develop Chagas cardiomyopathy, a progressive myocardial disease. CMD characterized by structural capillary abnormalities, autonomic imbalance, and immune-mediated endothelial inflammation is a recognized hallmark of early and advanced Chagas heart disease, often precedes regional wall motion abnormalities, and occurs independently of epicardial coronary artery disease. TTDE allows non-invasive evaluation of CFVR, typically assessed in the distal LAD artery using color and spectral Doppler during pharmacological stress (e.g., adenosine, dipyridamole, or high-dose dobutamine). A blunted CFVR (typically <2.0) reflects impaired microcirculatory function and has been correlated with higher burdens of myocardial fibrosis (detected via cardiac magnetic resonance), elevated ventricular arrhythmias, and worse NYHA functional class in chronic Chagas cardiomyopathy [71]. However, the use of non-invasive CFVR assessment for predicting disease evolution requires further validation in prospective studies within Chagas populations.

### 3.4. Myocardial Bridges

In our daily clinical practice, the assessment of CFVR by TTDE using vasodilators in patients with myocardial bridges consistently yields results greater than 2.0. However, evidence has shown that CFV measurement during DOBSE provocation provides a more accurate functional evaluation of myocardial bridging compared to adenosine. Non-invasive CFV during dobutamine provocation appears to be a valuable tool to determine the functional severity of muscular bridges. A transthoracic CFVR cut-off of ≤2.1 measured at a high-dose dobutamine stage may be adequate for detecting myocardial ischemia in patients with isolated bridges [72].

### 3.5. Obesity

CFVR measured by TTDE provides significant prognostic stratification in obese patients, identifying individuals at elevated cardiovascular risk independently of body mass index (BMI) and traditional risk factors [7,73]. While noninvasive stress echocardiography (SE) primarily targets regional wall motion abnormalities to rule out obstructive epicardial coronary artery disease, it often overlooks subclinical vascular impairment. In overweight and obese individuals with preserved left ventricular ejection fraction (LVEF < 505), CMD quantified by a reduced CFVR (CFVR < 2.0) in the LAD serves as an early indicator of systemic cardiovascular vulnerability. In large prospective multicenter cohorts, impaired CFVR was identified in over 20% of patients despite a negative stress study for inducible ischemia. Importantly, CMD stands as a powerful and independent predictor of long-term overall mortality after adjusting for traditional metabolic comorbidities and demographic factors [73]. Incorporating functional microvascular assessment alongside conventional wall motion analysis enhances long-term risk stratification, guiding therapeutic decision-making in high-risk metabolic cohorts.

### 3.6. Diabetes Mellitus

In the context of diabetes, CFVR provides prognostic information that is independent of the severity of CAD or the degree of glycemic control. Even in patients whose coronary arteries do not show significant obstruction, a reduced CFVR can predict an increased risk of cardiovascular mortality. The ability of CFVR to identify high-risk individuals among diabetic patients underscores its value in guiding early intervention and aggressive management strategies. A preserved CFVR off-therapy is associated with better survival and yearly event rates comparable to those of non-diabetic patients [74].

In our laboratory, we evaluated the outcomes of 651 patients with a normal wall motion response during stress echocardiography. Normal CFVR was recorded in 523 patients, while reduced CFVR was found in 128 patients. During a mean follow-up period of 34.6 ± 18 months, 48 major events occurred: 25 patients (4.8%) in the normal CFVR group and 23 patients (18%) in the reduced CFVR group. Event-free survival was significantly different between patients with normal versus abnormal CFVR (*p* < 0.0001). This study revealed a highly specific nuance: diabetic patients with a normal CFVR had a favorable prognosis, similar to non-diabetics. Diabetes only significantly worsened the prognosis (*p* = 0.05) if the patient already presented with an abnormal CFVR [75].

### 3.7. Final Considerations 

At this point, a reasonable question arises: if evaluating coronary flow and CFVR using TTDE provides such valuable diagnostic insights, why is this technique not more widely adopted in echocardiography laboratories worldwide? The answer is multifaceted and open to debate. Overcoming inertia will require dispelling several common myths. Contrary to widespread perception, the technique is neither time-consuming nor technically complex; it adds only a few minutes to standard SE, has a short learning curve, and is fully feasible in obese patients or those with suboptimal acoustic windows [9]. Furthermore, it is not limited to vasodilators, as alternative stressors—such as dobutamine or exercise—can also be applied. It is essential to emphasize that CFVR integrates the physiological response of the entire coronary vascular bed; thus, an impaired CFVR is not exclusive to obstructive epicardial coronary artery disease but heavily relies on the structural and functional status of the coronary microvasculature. However, a preserved or normal CFVR value reliably excludes the presence of hemodynamically significant epicardial coronary stenosis. Among available techniques, TTDE -based CFVR determination represents the most cost-effective and ecologically sound option; although constrained by its inability to quantify flow, its clinical assessment remains pivotal, particularly in ADA (Table 5). In our daily clinical practice, this approach has proven invaluable for decision-making and serves as an ideal framework for assessing various conditions and therapeutic interventions. We fully endorse the position of the European Association of Echocardiography Expert Consensus Statement: “Adding CFVR evaluation of the LAD by TTDE introduces another critical dimension to stress echocardiography.” [31]. CFVR is now integrated into the comprehensive ABCDE stress echocardiography protocol: **A** for Asynergy (regional wall motion abnormalities), **B** for B-lines, **C** for Contractile reserve, **D** for Doppler-based CFVR, and **E** for ECG-based heart rate reserve [7,76]. A blunted CFVR response carries independent prognostic significance beyond inducible wall motion abnormalities for predicting adverse outcomes across various cardiac disorders. While ongoing technological refinements and multicenter trials will further shape its application, this technique is undoubtedly established in modern practice [29].

## 4. Conclusions

- Transthoracic coronary flow velocity assessment is a simple, feasible, safe, and reproducible approach to evaluating the functional status of the coronary circulation in diverse cardiovascular conditions.
- CFVR measurement provides substantial guidance in risk stratification; a reduced CFVR—whether elicited by adenosine, dipyridamole, dobutamine, or exercise—is a robust, independent predictor of MACE.
- Clinicians should note that a blunted CFVR cannot definitively differentiate severe epicardial stenosis (macrovascular CAD) from microvascular dysfunction. Nonetheless, integrating clinical evaluation, transient regional wall motion abnormalities during SE, and markedly reduced CFVR levels can facilitate the differential diagnosis.
- Routine clinical implementation of CFVR should be expanded to better tailor aggressive risk-factor modification in high-risk patient.

## Figures and Tables

**Figure 1:**
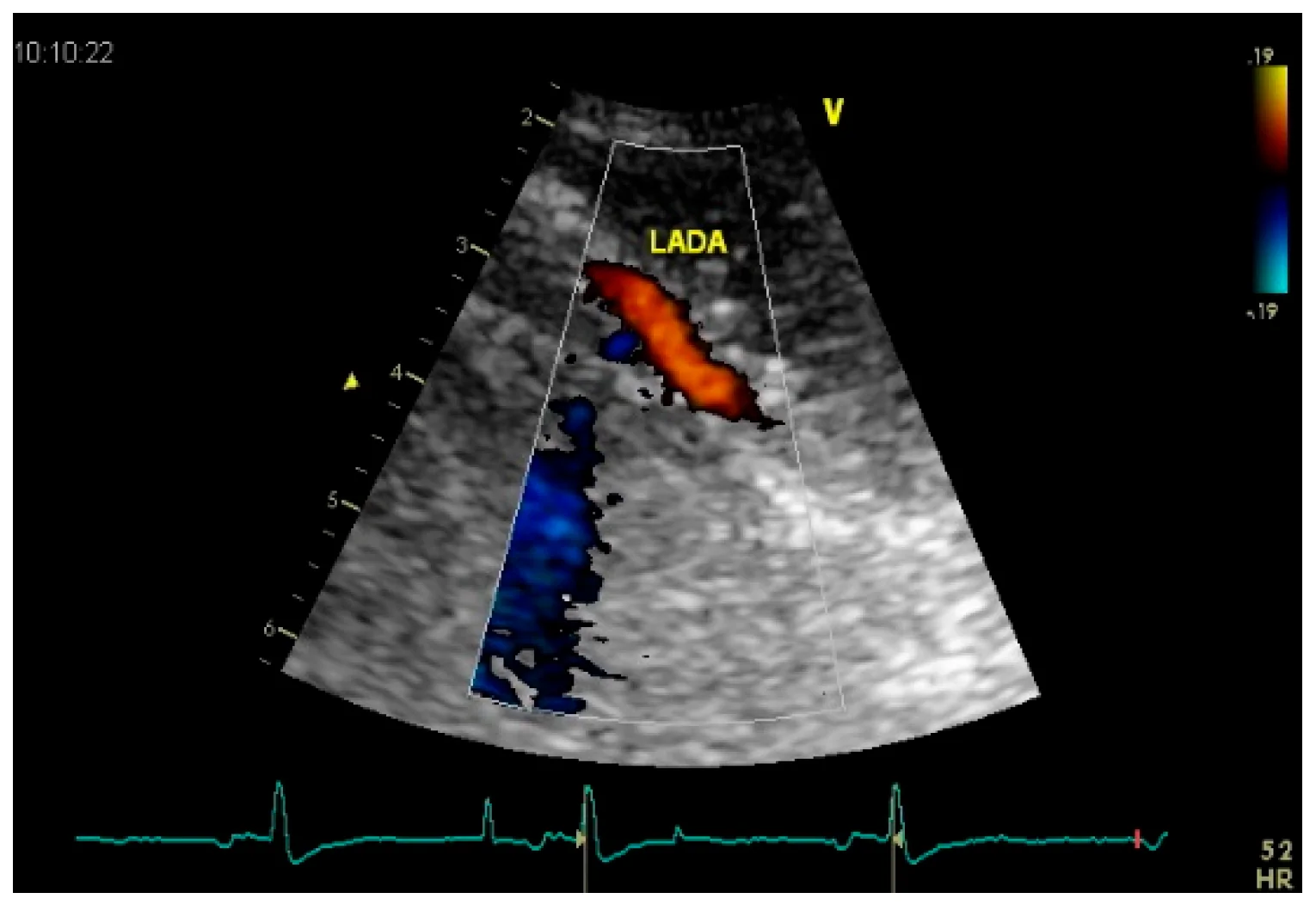
Color Doppler imaging of the distal left anterior descending artery (LAD) coronary artery in the anterior interventricular groove.

**Figure 2:**
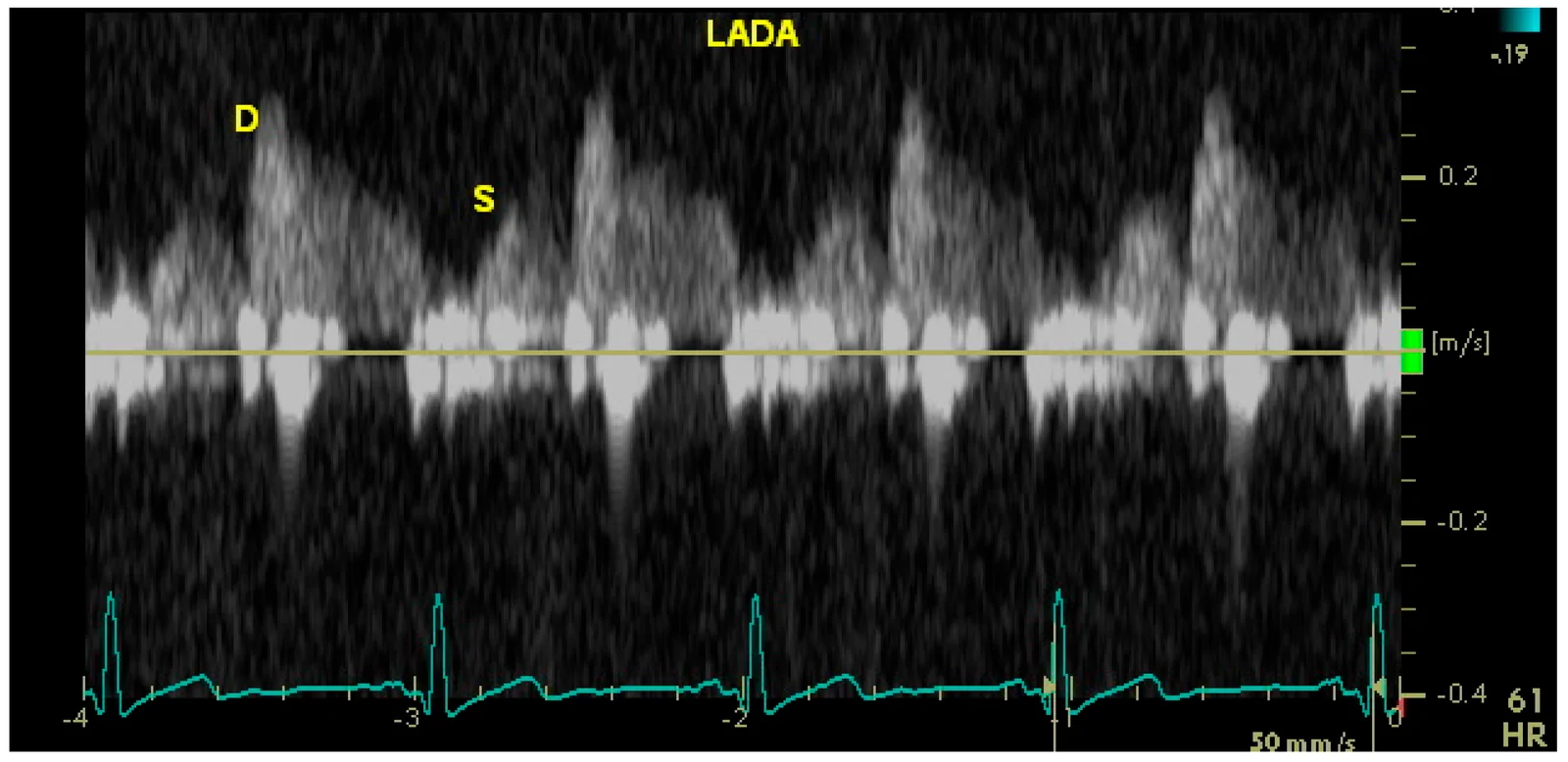
Pulse Doppler velocity flow of the distal left anterior descending artery (LAD) coronary artery with the typical biphasic coronary flow pattern.

**Figure 3:**
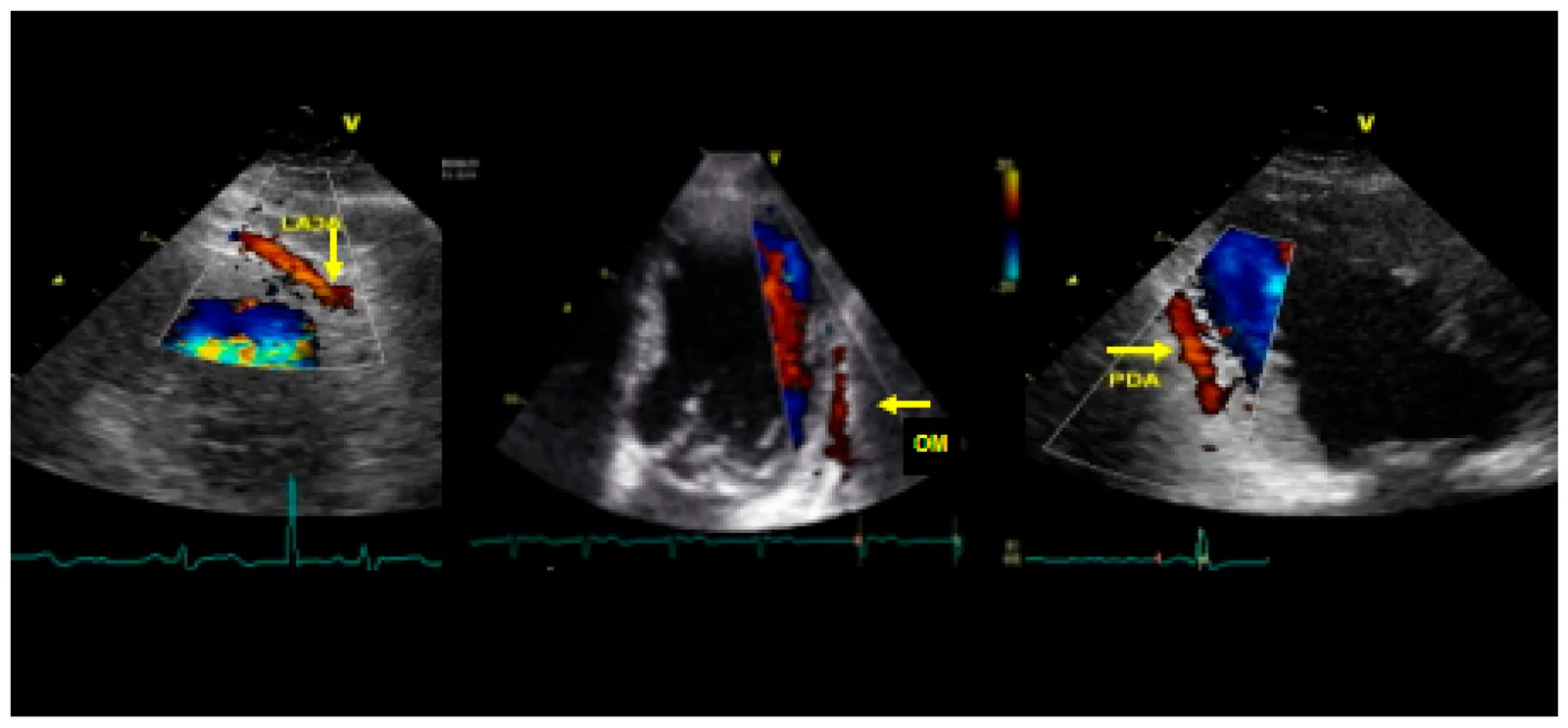
Left: Color Doppler imaging of the distal left anterior descending artery (LAD) coronary artery. Middle: The obtuse marginal (OM) branch of the left circumflex artery. Right: Imaging of the posterior descending coronary (PD) artery arising from a dominant right coronary artery.

**Figure 4:**
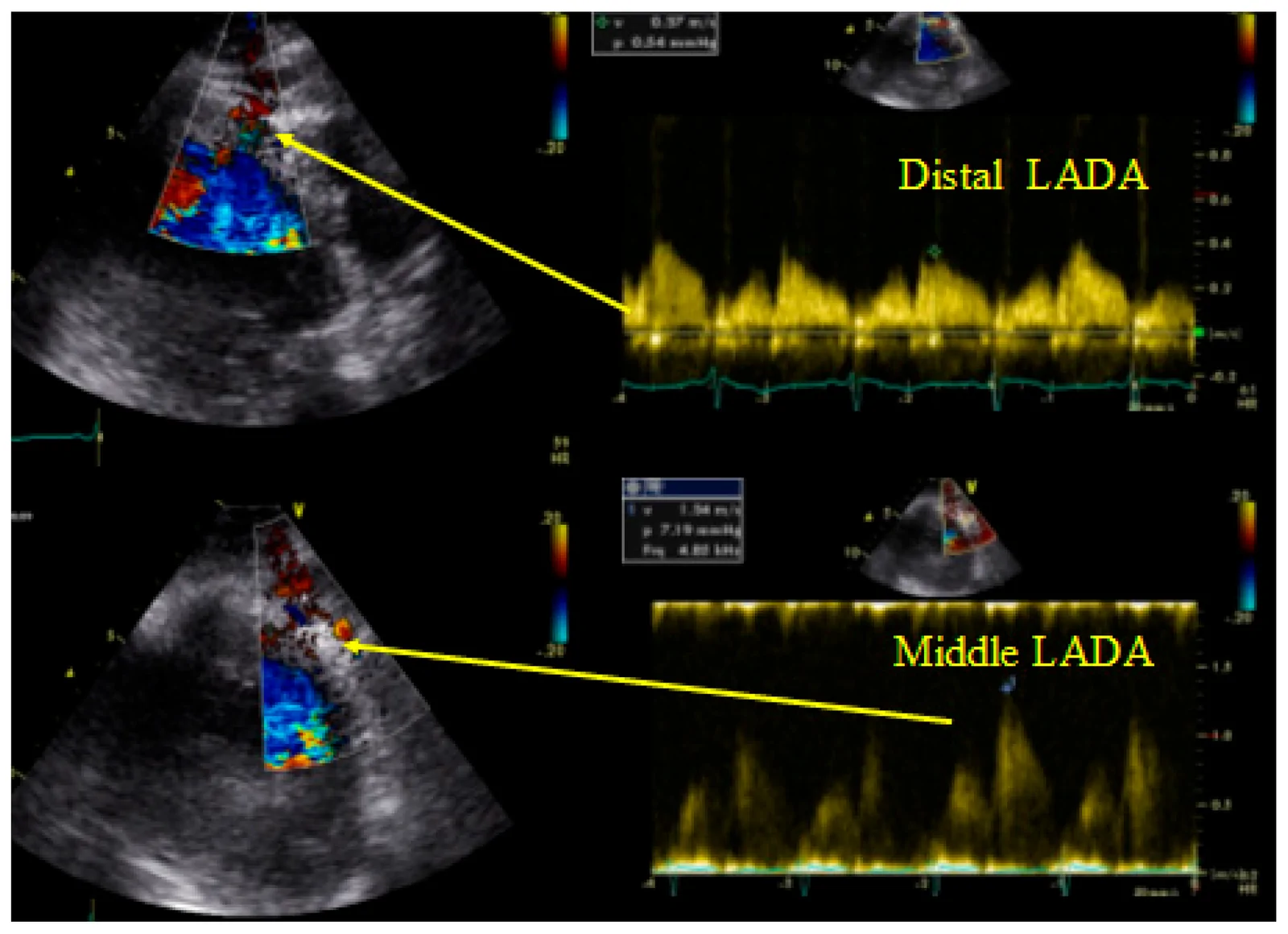
Color Doppler image of an asymptomatic patient. **Upper panel:** Diastolic flow velocity in the distal LAD is 37 cm/s, compared to 134 cm/s in the adjacent middle LAD, yielding a distal-to-proximal ratio of 0.27. A ratio **≤ 0.5** is predictive of significant stenosis. Coronary angiography confirmed a 95% stenosis in this patient.

**Figure 5:**
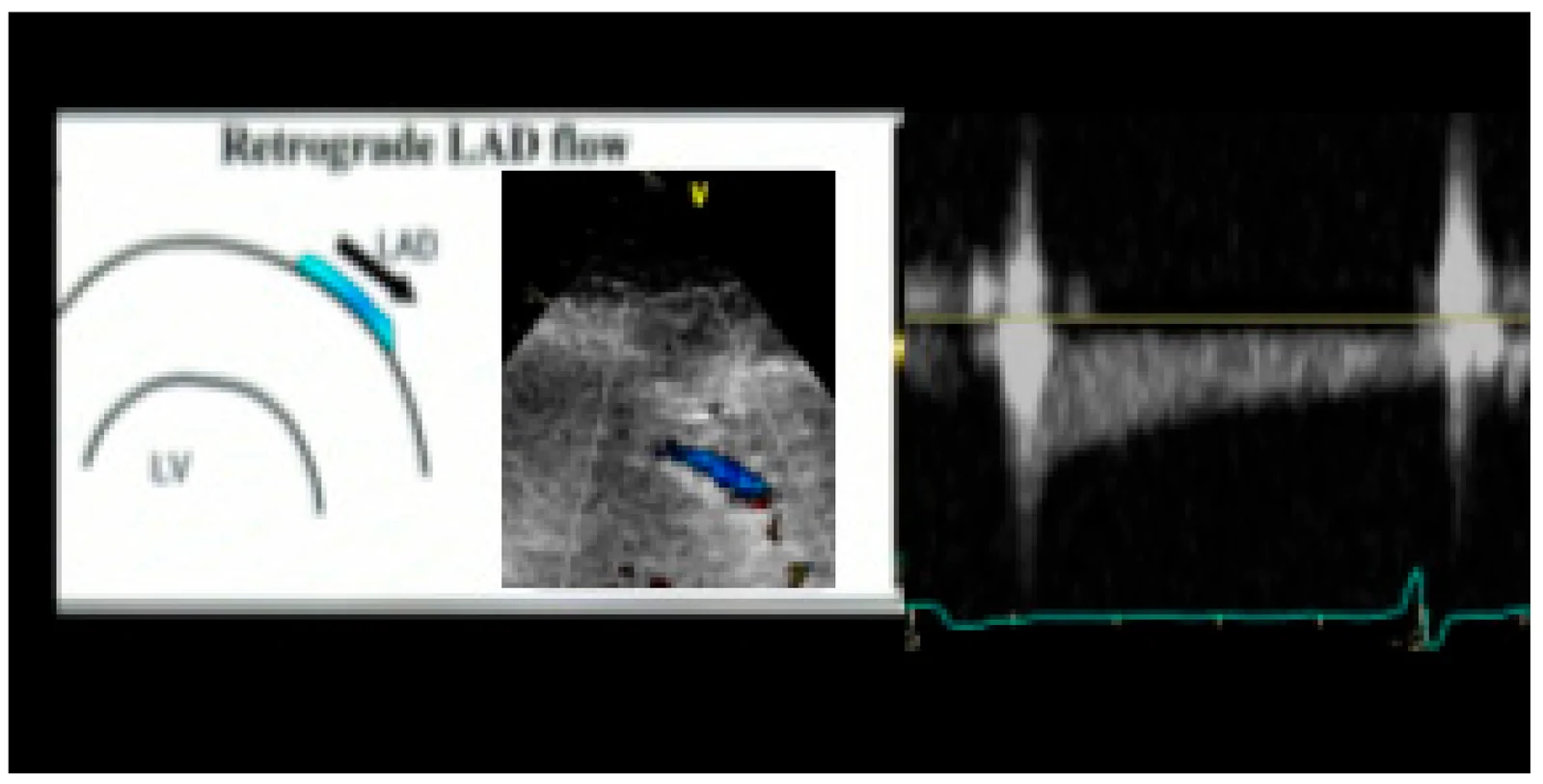
Color Doppler image showing retrograde flow in the left anterior descending (LAD) coronary artery due to total occlusion. The position of the pulsed Doppler signal within the interventricular groove is key to the differential diagnosis with a perforator branch.

**Figure 6:**
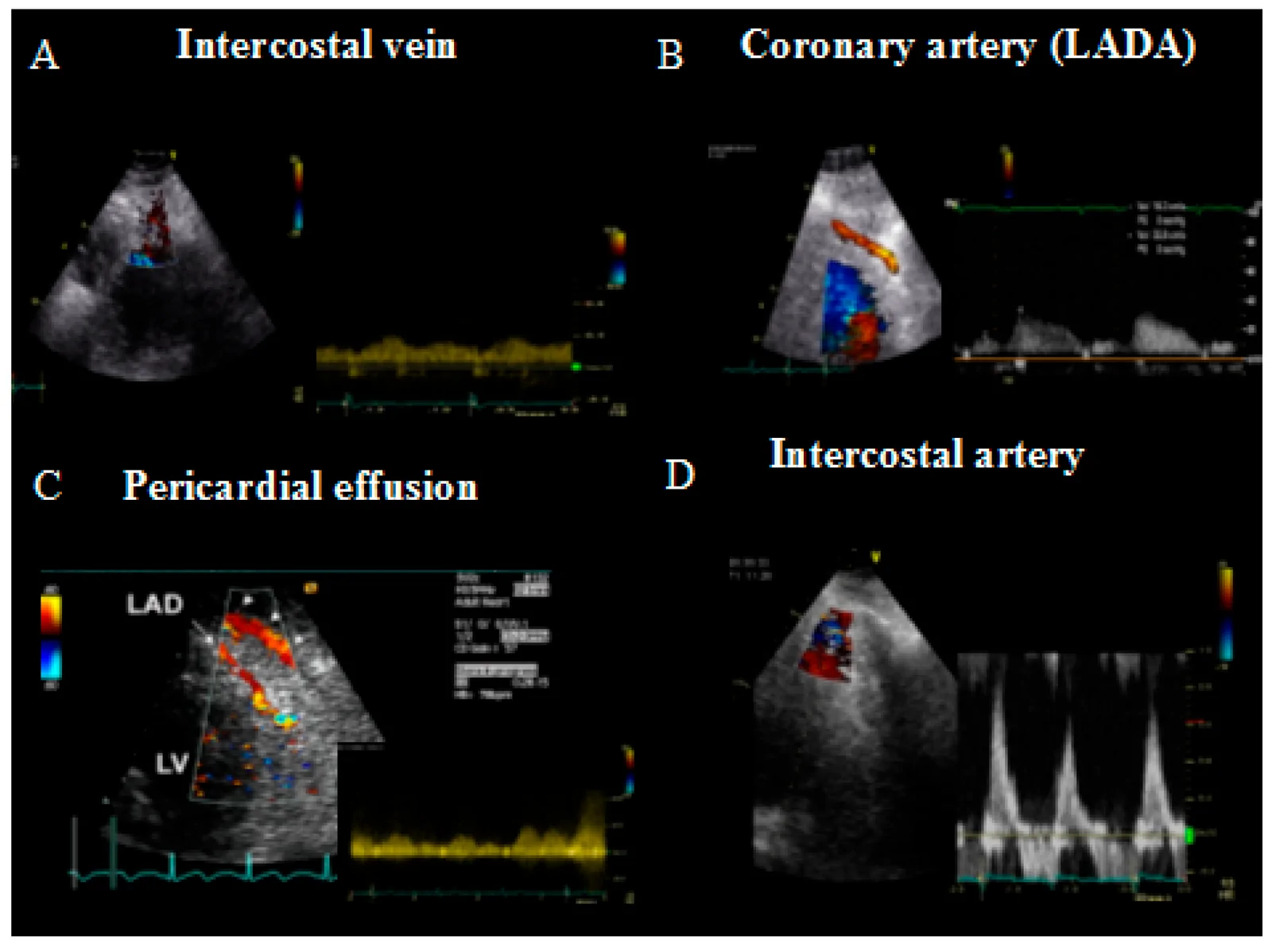
Distinctive flow characteristics of normal coronary flow vs. intercostal artery, pericardial space, and intercostal flow. (**A**) Intercostal vein: Flow with respiratory variation. (**B**) Coronary artery: Typical biphasic pattern. (**C**) Pericardial effusion: Heterogeneous flow. (**D**)Intercostal artery: Predominant systolic flow.

**Figure 7:**
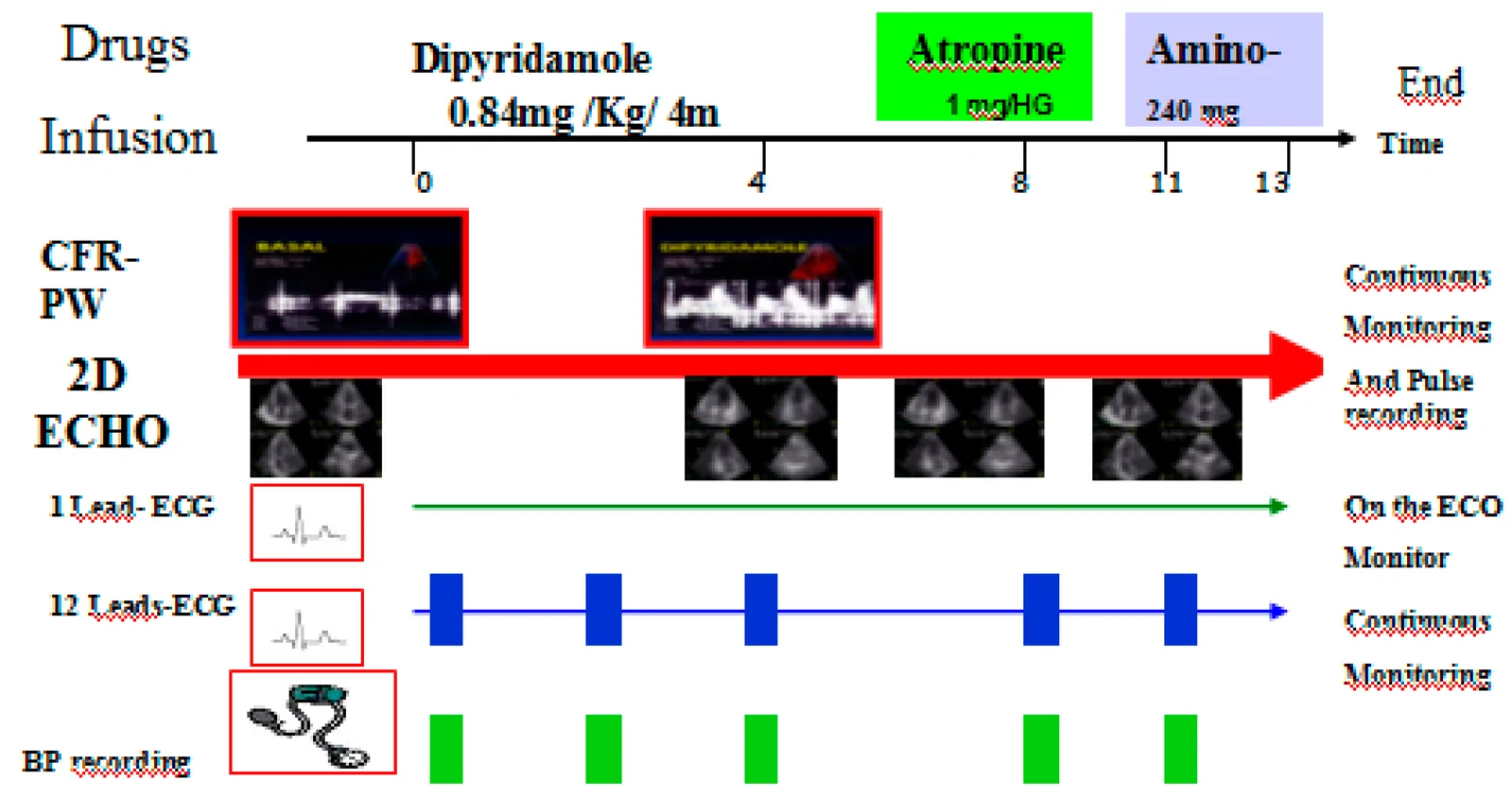
Current protocol of dual dipyridamole stress echocardiography. 2D echo = 2-dimensional echocardiogram. Abbreviations: BP = blood pressure; ECG = electrocardiogram; PW = pulse wave. Modified from the European Guidelines, European Journal of Echocardiography 2008 9415-37 [31].

**Figure 8:**
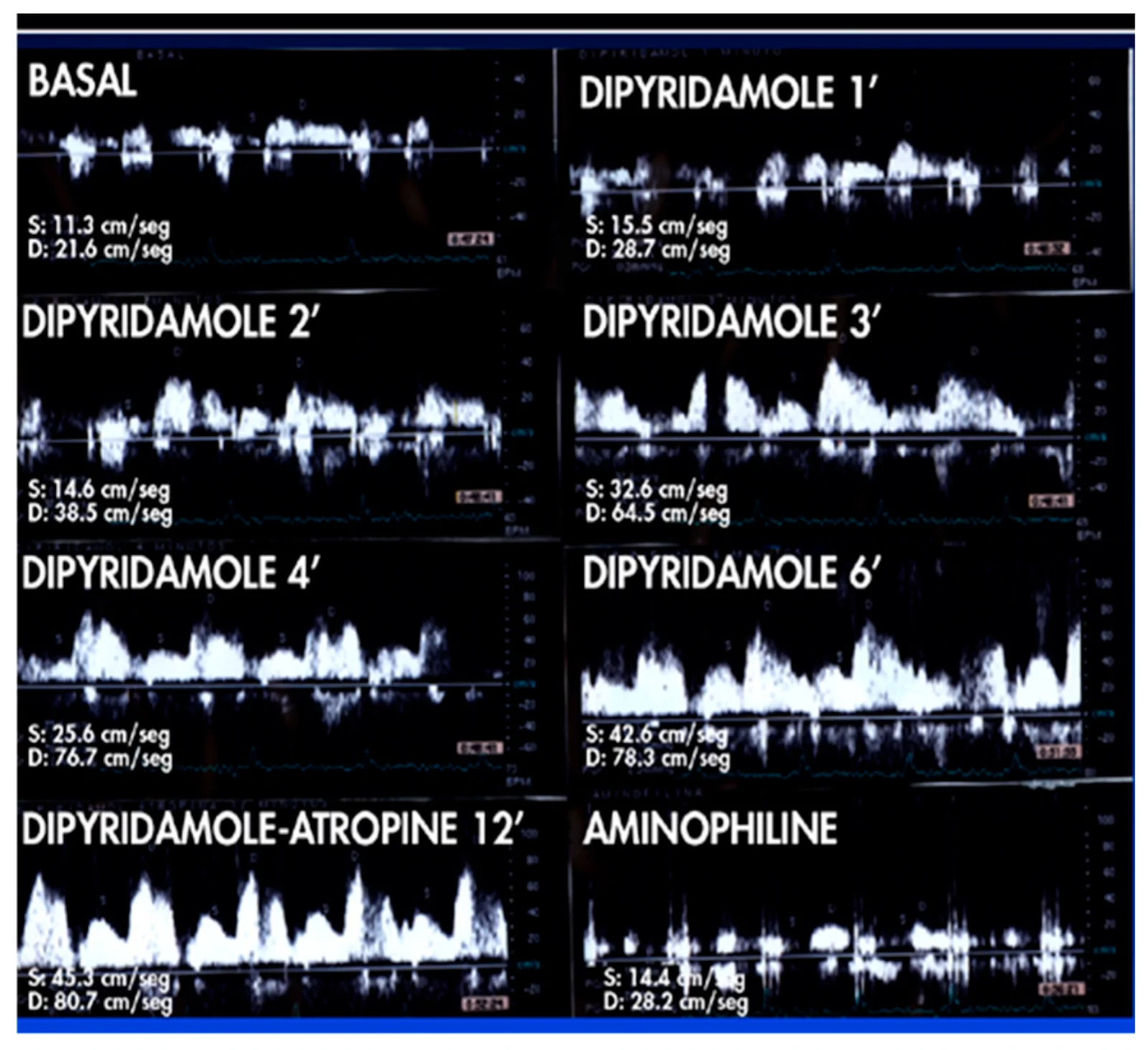
Continuous Analysis or the CFV of the LAD by TTDE during Dipyridamole infusion.

**Figure 9:**
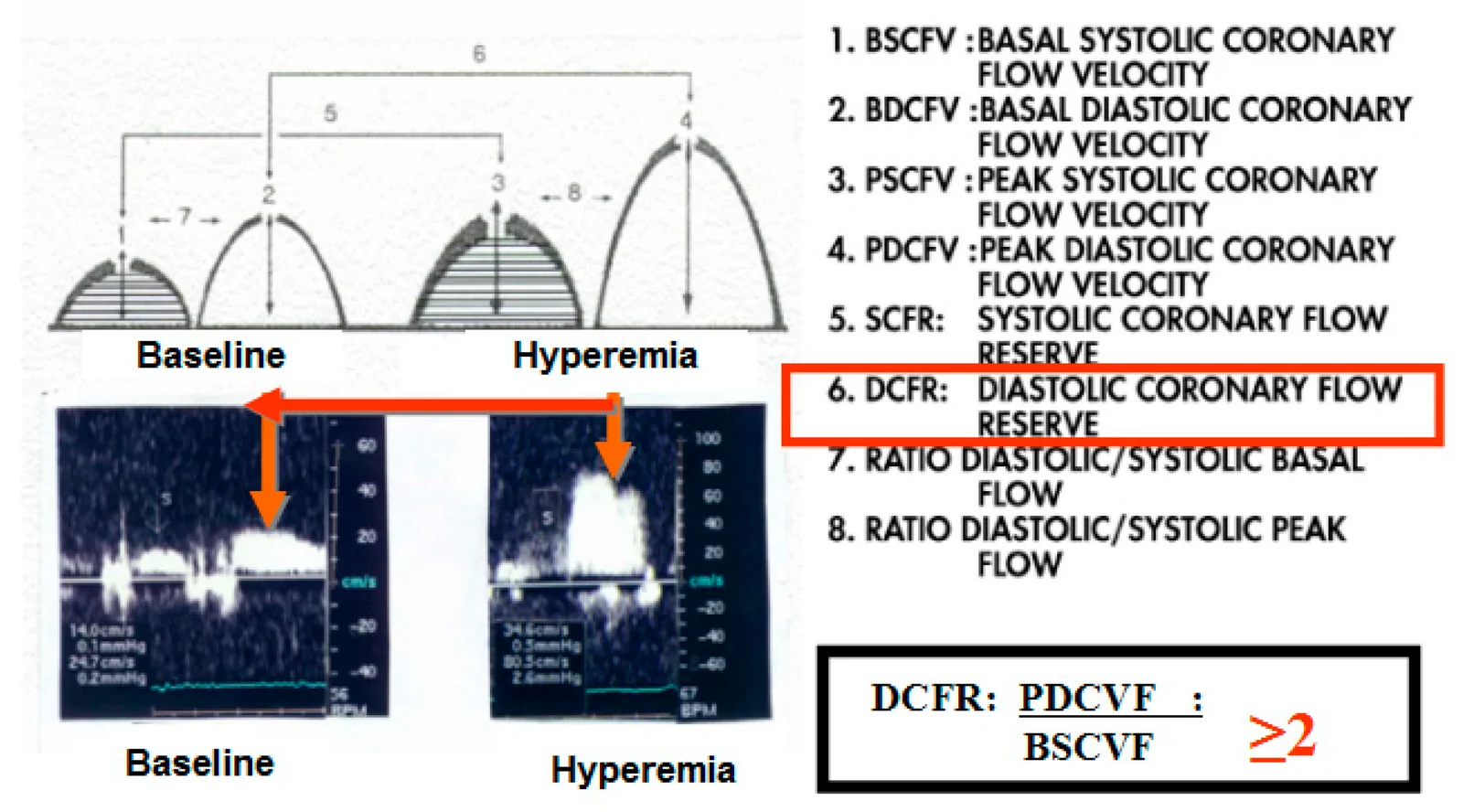
Coronary blood flow velocity reserve was defined as the ratio between hyperemic and baseline peak diastolic coronary flow. CFVR value ≥ 2 is considered normal.

**Figure 10:**
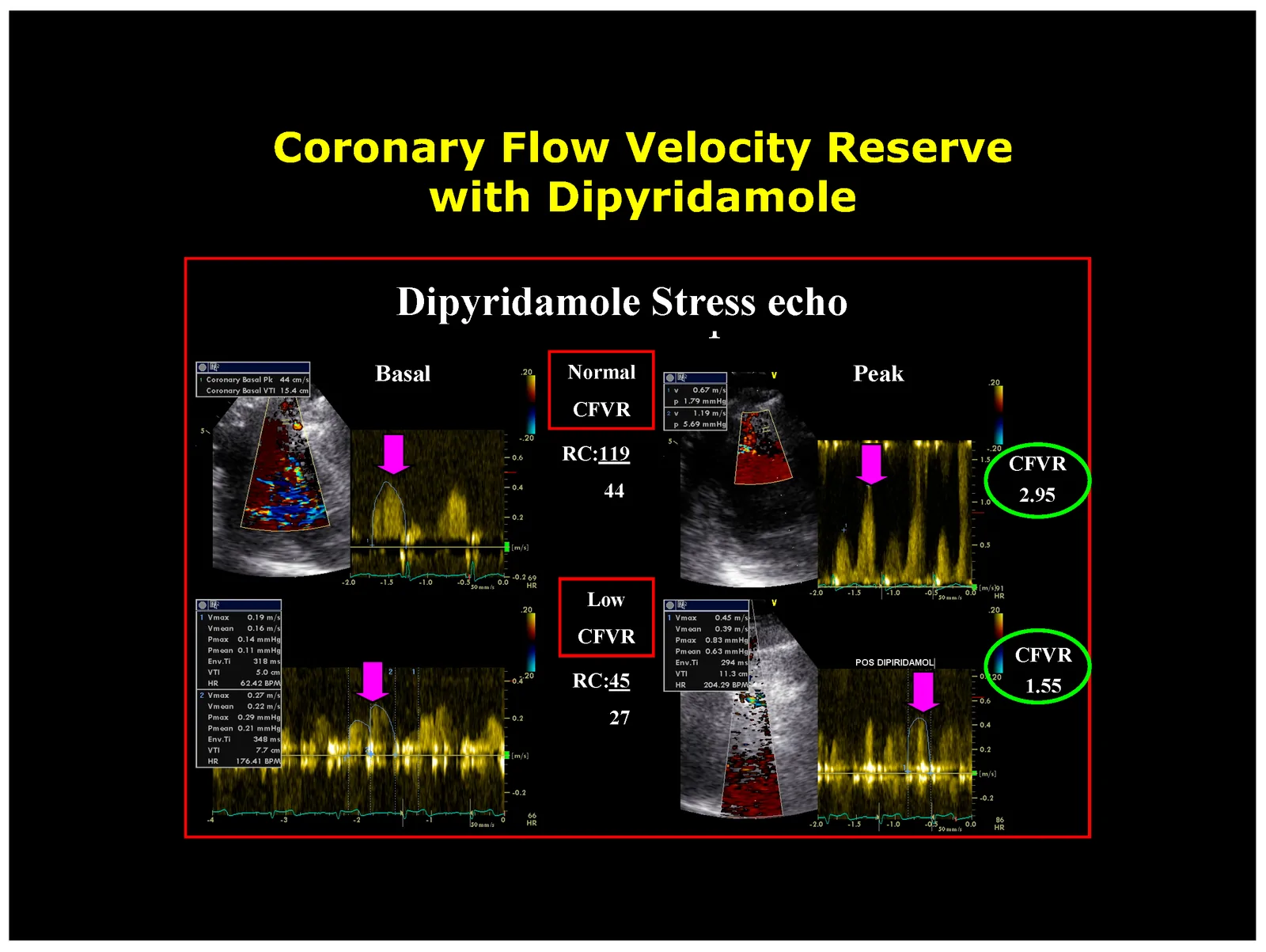
Coronary Flow Velocity Reserve (CFVR) evaluation via DIPSE. (**Top**) Typical example of normal CFVR demonstrating a significant increase in Doppler peak diastolic flow velocity from baseline to peak hyperemic flow in the left anterior descending artery (LAD). (**Bottom**) Representative example of abnormal CFVR.

**Figure 11:**
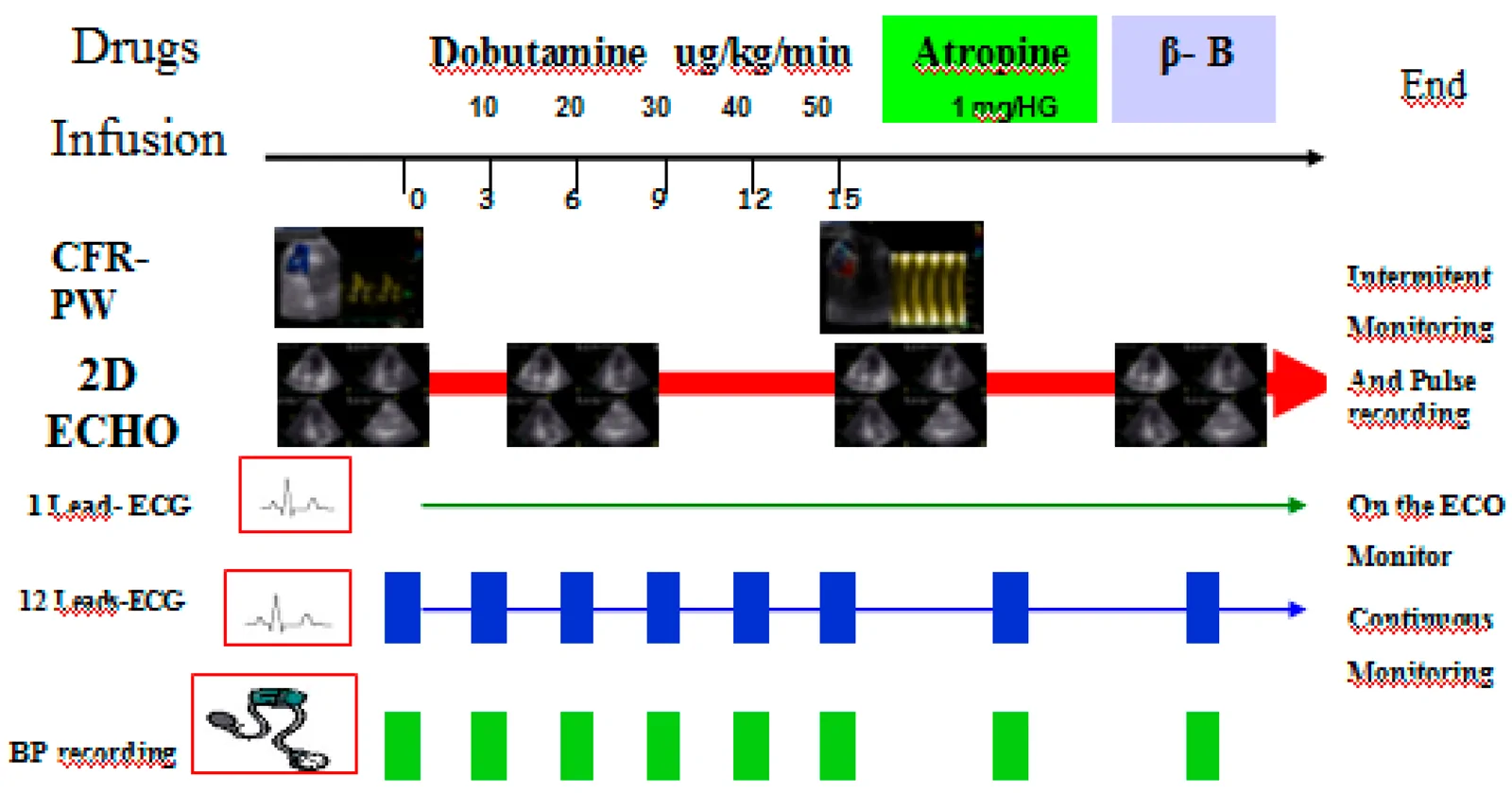
Current protocol of dual dobutamine stress echocardiography. Abbreviations: 2D echo = 2-dimensional echocardiogram; BP = blood pressure; ECG = electrocardiogram; PW = pulse wave. Modified from the European Guidelines, European Journal of Echo-cardiography 2008 9415-37.

**Figure 12:**
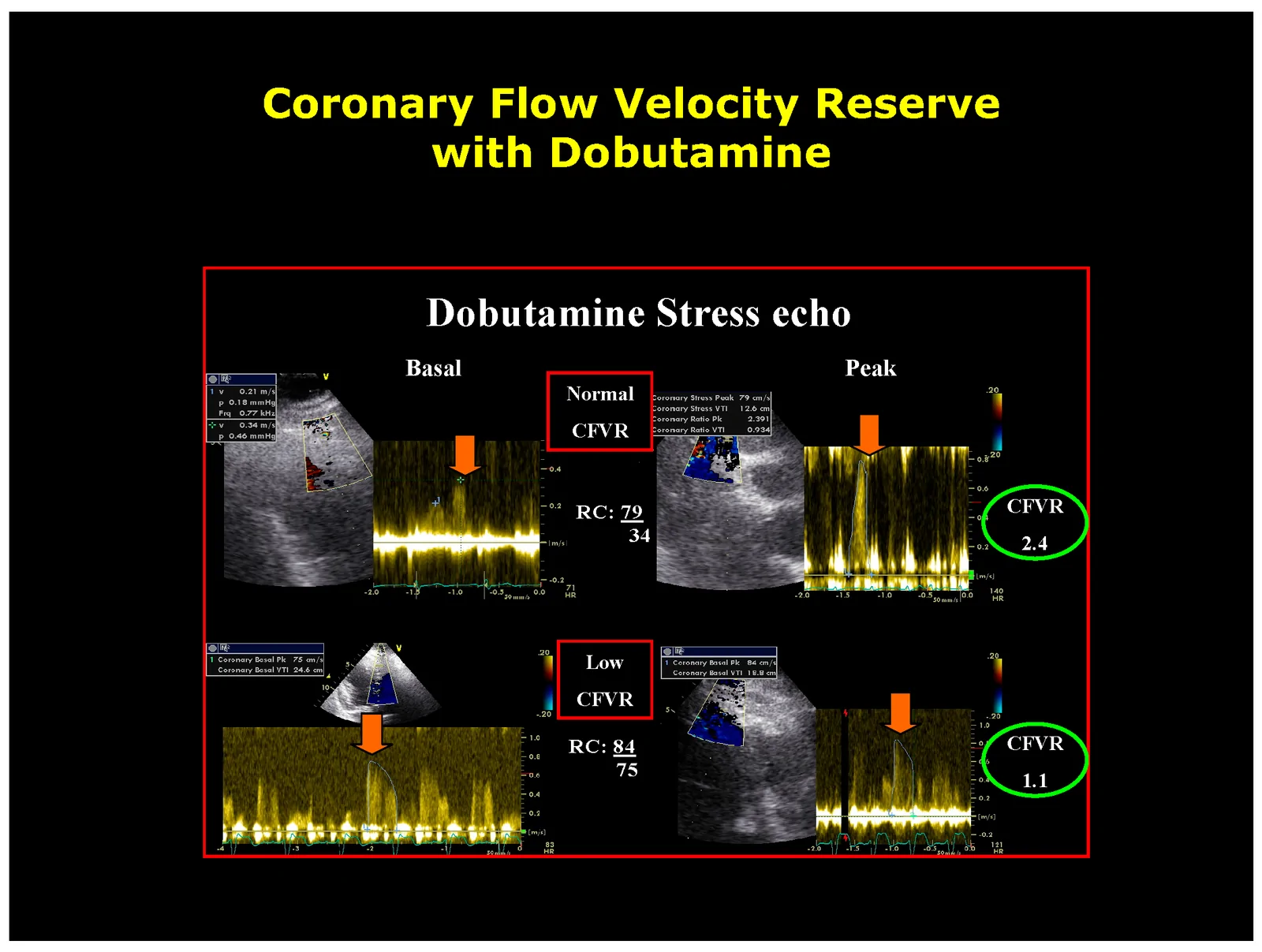
Top: A typical example of normal CFVR with normal wall motion during Dobutamine stress echocardiography, showing a significant increase in LAD peak diastolic Doppler flow velocity from baseline to peak stress. Bottom: An abnormal CFVR response.

**Figure 13:**
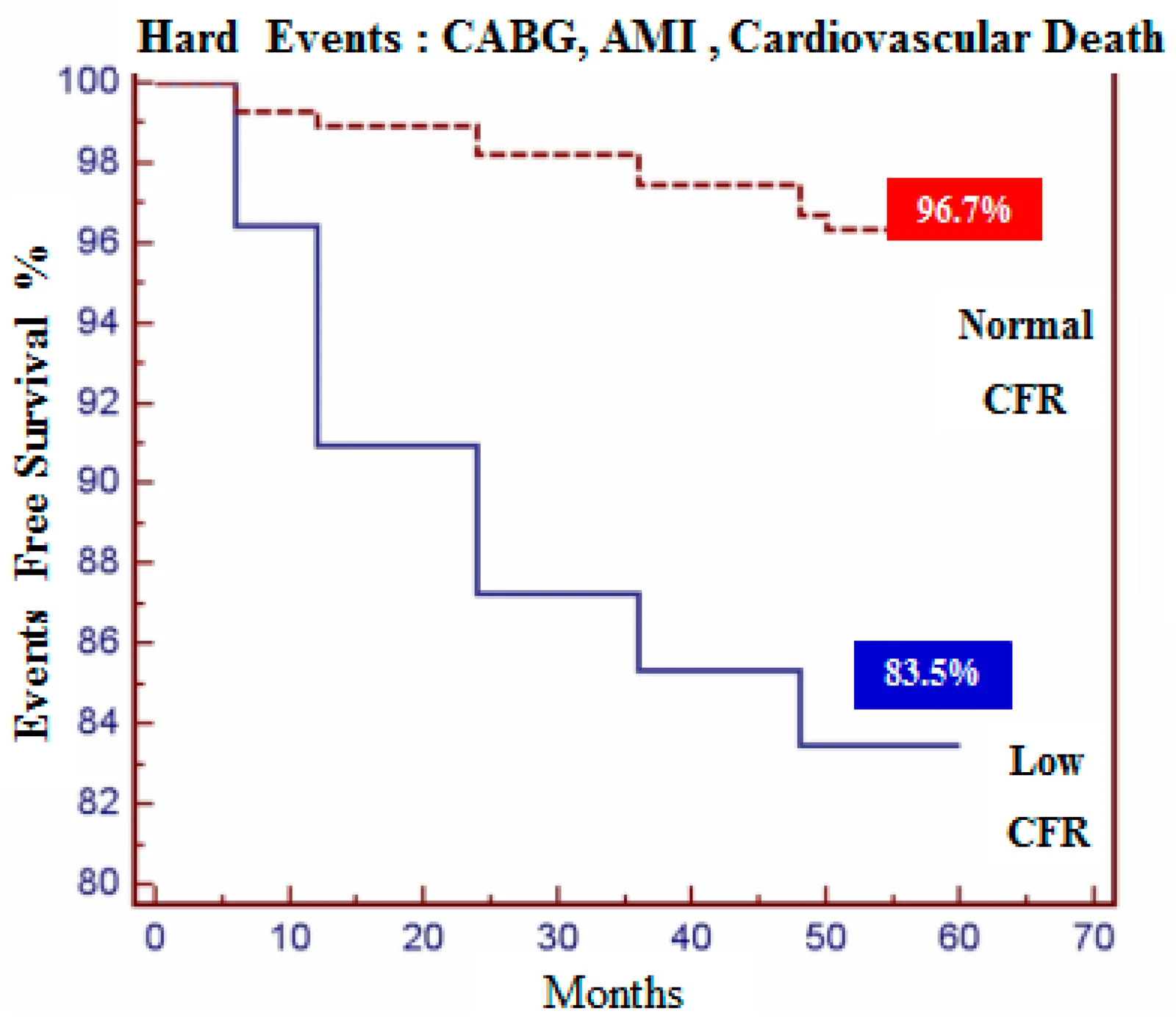
Kaplan–Meier survival curves show that event-free survival estimated at 45 ± 21 months for the group with normal CFVR was significantly higher than that of the group with reduced CFVR (96.7% vs. 83.5%, Log-rank test = 0.0001). Abbreviations: CABG: Cardiac Artery Bypass Graft, AMI: Acute myocardial Infarction.

**Figure 14:**
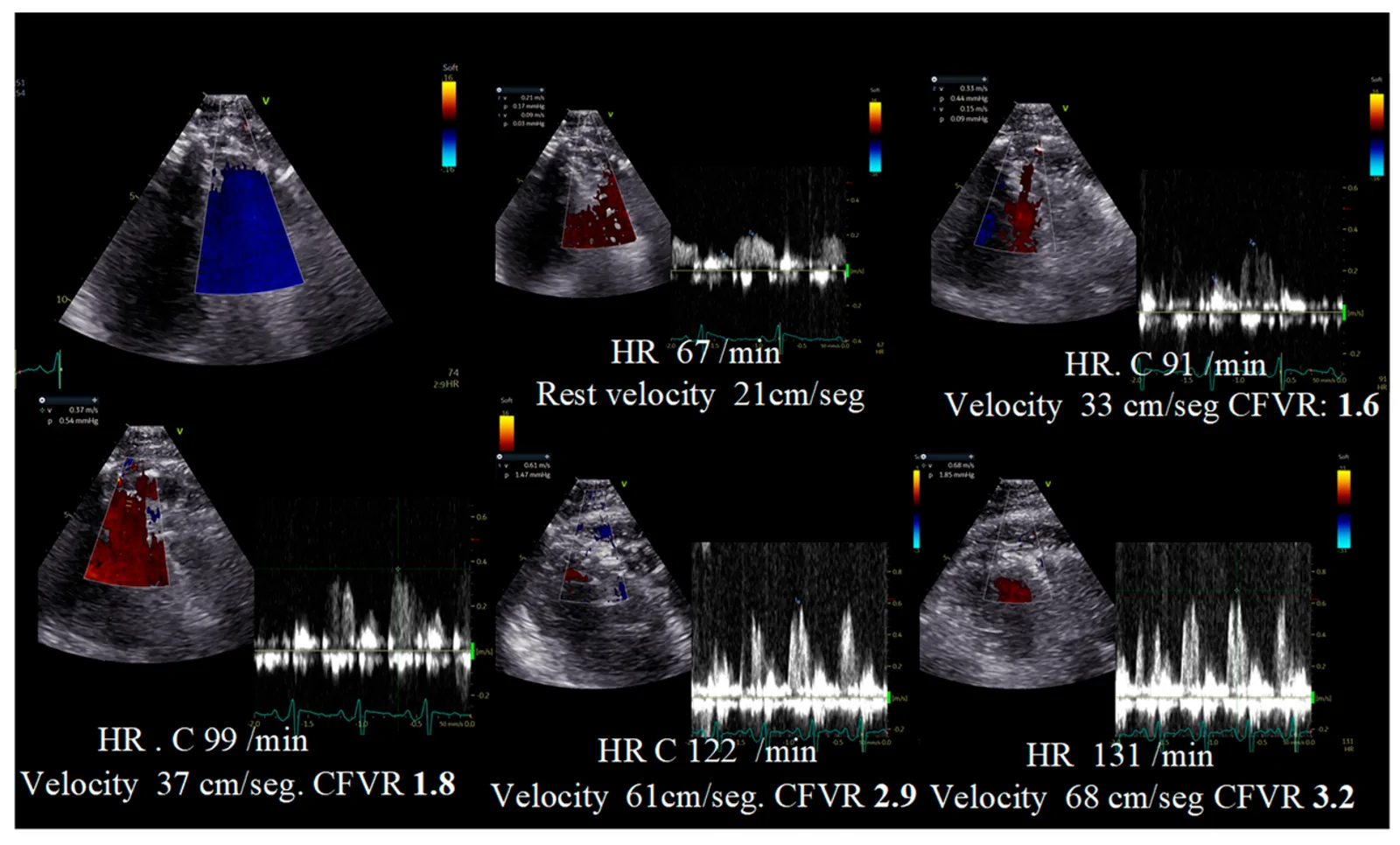
Coronary flow velocities during supine stress echocardiography. HR: Heart rate.

**Figure 15:**
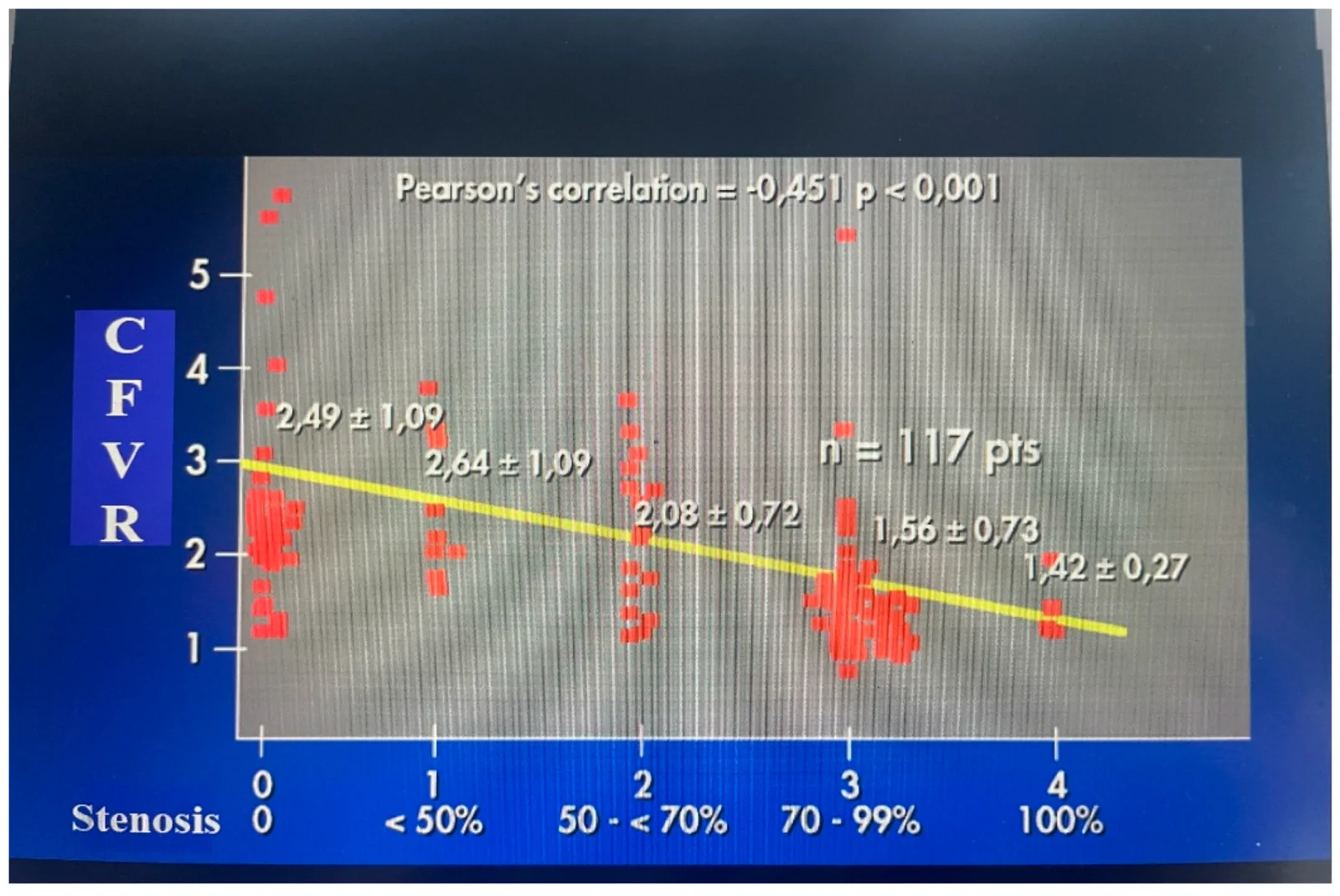
Correlation between non-invasive assessment of CFVR and stenosis severity of LAD [45].

**Table 1:** Feasibility of simultaneous assessment of CFVR and wall motion analysis in the studies reported in the review.

Study/Reference	Year	Patients (n)	Stress Agent/Protocol	Vessel/Feasibility (%)	Contrast Used
**Lowenstein J et al.** [10]	2000	170	Dipyridamole	LAD: 95	No
**Rigo F et al.** [5]	2005	658	Dipyridamole	LAD: 98 RCA: 53 LCX: 43	Yes
**Nihoyoshi Y et al.** [11]	2003	110	Dipyridamole	LAD: 92	Yes
**Chirillo F et al.** [12]	2004	110	Dipyridamole	LAD: 85	No
**Ascione L et al.** [13]	2006	159	Dipyridamole	LAD: 92	Yes
**Meimoun P et al.** [14]	2008	47	Dobutamine Adenosine	LAD: 87 (Dobutamine) LAD: 91 (Adenosine)	Yes
**Forte E et al.** [15]	2011	33	Dobutamine	LAD: 94	No
**Takeuchi M et al.** [16]	2003	274	Dobutamine	LAD: 85	Yes
**Ahmari SA et al.** [17]	2006	67	Dobutamine	LAD: 97	Yes
**Florenciano-Sánchez R et al.** [18]	2005	130	Dobutamine	LAD: 84	No
**Hyodo E et al.** [19].	2010	175	Adenosine	LAD: 95 RCA: 85 LCX: 81	Yes
**Vegsundvåg J et al.** [20]	2011	108	Adenosine	LAD: 97 RCA: 75 LCX: 63	No
**Zagatina A et al.** [21]	2016	689	Exercise	LAD: 81	No

Abbreviations: CFVR: coronary flow velocity reserve; LAD: left anterior descending artery; RCA: right coronary artery; LCX: left circumflex artery.

**Table 2:** Diagnostic and Risk Stratification Framework Combining Wall Motion and Coronary Flow Velocity Reserve.

Clinical/Diagnostic Phenotype	Regional Wall Motion (WMA)	Coronary Flow Velocity Reserve (CFVR)	Primary Pathophysiologic Substrate	Clinical Interpretation & Risk Profile
**Normal/Low Risk**	Normal (No inducible dyssynergy)	Preserved (≥2)	Intact epicardial conductance and microvascular vasodilator capacity	Excellent long-term outcome; minimal cardiac event risk (<1%/year).
**Coronary Microvascular Dysfunction (CMD)**	Normal (No inducible dyssynergy)	Impaired (<2.0) *	Impaired arteriolar vasodilation or structural microvascular remodeling	Heightened cardiovascular risk despite non-obstructive epicardial arteries; warrants targeted risk-factor modification.
**Obstructive Epicardial CAD (Ischemic)**	Abnormal (Inducible dyssynergy in ≥1 territory)	Impaired (<2.0) *	Hemodynamically significant epicardial stenosis (±concomitant microvascular impairment)	High ischemic burden and elevated MACE risk; consider invasive coronary angiography and revascularization.
**Non-Ischemic Cardiomyopathy/Metabolic Phenotype**	Normal or Global LV Dysfunction (No regional ischemia)	Impaired (<2.0) *	Microvascular rarefaction, interstitial fibrosis, or elevated baseline diastolic velocities	Adverse remodeling and increased long-term heart failure/mortality risk across diverse non-ischemic pathologies.
**Severe Multivessel CAD**	Multiple WMAs (Inducible dyssynergy in multiple territories)	Severely Impaired (<1.5)	Severe multi-vessel epicardial obstruction	Very High Risk/Critical Ischemic Burden; urgent invasive coronary angiography and consideration for revascularization required.

* Note: A threshold of <1.89$ may be applied when evaluating CFVR during exercise stress echocardiography (ESE) protocols.

**Table 3:** Relation between CFVR value and event chance during a follow-up of 46 ± 24 months [38].

CFVR	Event Chance (OR)	IC (95%)	*p*
≥2	1.08		NS
Between 2 and 1.65	3.84	1.22–12.13	0.02
Between 1.64 and 1.47	4.57	1.14–18.38	0.03
≤1.47	5.06	1.21–21.16	0.02

**Table 4:** Clinical Scenarios Where Flow Velocity and Coronary Flow Velocity Reserve (CFVR) Measurements Have Proven Useful.

Measurement of Baseline Flow Velocity	Measurement of CFVR
Diagnosis of high probability of severe coronary stenosis (the diastolic velocity of the flow is decreased compared to the systolic velocity (D/S < 1.5)) [24].	Diagnosis of significant coronary stenosis (CFVR < 2 and Dual imaging (and wall motion) during stress echocardiography [7,8,9,22,36,42,43]
Diagnosis of coronary stenosis (aliasing with >1 mt/sec velocity at the site of stenosis, or a high gradient between two segments of a coronary artery) [23].	Follow-up after coronary angioplasty and stenting and other revascularization procedures [19,44].
Resting CFV ≥ 32 cm/s was identified as the optimal cutoff and was independently associated with mortality [22].	Evaluation of coronary stenosis of intermediate severity [45,46].
Diagnosis of coronary occlusion (reverse diastolic flow) [25].	Patients with LBBB of unknown ethology [47,48].
	Evaluation of coronary microcirculation (ANOCA–INOCA, angina or ischemia with normal coronary arteries [39,49,50,51,52,53]
	Evaluation of valvular heart diseases [54,55,56], dilatated myocardiopathy [57,58], hypertrophic cardiomyopathy [59,60]. Heart failure [61,62], heart transplantation [63,64], Rheumatic diseases [65,66,67], Kawasaki [68,69], Takosubo [70], Chagas disease [71], muscular bridges [72], obesity [73], diabetes mellitus [74,75].

**Table 5:** Comparative analysis of common diagnostic imaging modalities for CFVR assessment: Strengths and limitations.

Modality	Availability	Costs	Radiation Exposure	Repeatability	Operador Dependency	Full CMV Function Assessment	Quantitative CBF Measure
TTDE	+++	+++	-	+++	-	-	-
PET	-	-	+++	+/-	++	+++	+++
CMR	+/-	-	-	+/-	++	+++	++
ICD	+/-	-	+	-	+	+	+

Modified from Del Buono MG et al. [77]. TTDE: transthoracic Doppler echocardiography. PET: Positron Emission Tomography. CMR: cardiac magnetic resonance. ICD: intracoronary Doppler. CMV Coronary microvascular. CBF: coronary blood flow. - poor for the item. + sufficient for the item. ++ good for the item. +++ very good for the item. The “+/-” notation represents “moderate performance”.

## Data Availability

No new data were created or analyzed in this study. Data sharing is not applicable to this article.

## Abbreviations

The following abbreviations are used in this manuscript:

CFVR	Coronary Flow Velocity Reserve
CFV	Coronary Flow Velocity
SE	Stress Echocardiography
MACE	Major Adverse Cardiovascular Events
TTDE	Transthoracic Doppler Echocardiography
LAD	Left Anterior Descending Artery
RCA	Right Coronary Artery
PDA	Posterior descending artery
LCx	Left Circumflex Coronary Artery
DIPSE	Dipyridamole Stress Echocardiography
DOBSE	Dobutamine Stress Echocardiography
ESE	Exercise Stress Echocardiography
CAD	Coronary Artery Disease
HF	Heart Failure
CMD	Coronary Microvascular Dysfunction
WMA	Wall Motion Abnormalities
CAV	Cardiac allograft vasculopathy
